# Genomic characterization of *Aliarcobacter* spp. from livestock in commercial and smallholder farms and from children in Ghana and Tanzania

**DOI:** 10.1016/j.onehlt.2026.101616

**Published:** 2026-10-13

**Authors:** Andreas E. Zautner, Ellis K. Paintsil, Charity Wiafe Akenten, Stephan Lorenzen, Linda Aurelia Ofori, Joyce Mbwana, Neyaz Ahmed Khan, John Andrea Lusingu, Joseph Kaseka, Daniel Minja, Samwel Gesase, Anna Jaeger, Maike Lamshöft, Achim J. Kaasch, Kwasi Obiri-Danso, Ralf Krumkamp, Jürgen May, Denise Dekker

**Affiliations:** a Institute of Medical Microbiology and Hospital Hygiene, Medical Faculty, Otto von Guericke University Magdeburg, 39120 Magdeburg, Germany; b Institute of Laboratory Medicine and Microbiology & Vaccination Centre, Klinikum Würzburg Mitte, Würzburg, Germany; c Kumasi Centre for Collaborative Research in Tropical Medicine (KCCR), South-End, Asuogya Road, 039-5028 Kumasi, Ghana; d Department of Theoretical and Applied Biology, Kwame Nkrumah University of Science and Technology, 039-5028 Kumasi, Ghana; e Department of Implementation Research, One Health Bacteriology Group, Bernhard Nocht Institute for Tropical Medicine (BNITM), Bernhard-Nocht-Str. 74, 20359 Hamburg, Germany; f Roger Williams Institute of Liver Studies, School of Immunology and Microbial Sciences, Faculty of Life Sciences and Medicine, King's College London, UK; g Bioinformatics Group, Bernhard Nocht Institute for Tropical Medicine, Hamburg, Germany; h National Institute for Medical Research (NIMR), Tanga, Tanzania; i Bernhard Nocht Institute for Tropical Medicine (BNITM), Bernhard-Nocht-Str. 74, 20359 Hamburg, Germany; j German Centre for Infection Research (DZIF), Partner site Hamburg-Lübeck-Borstel-Riems, 20359 Hamburg, Germany; k Tropical Medicine II, University Medical Center Hamburg-Eppendorf (UKE), 20251 Hamburg, Germany

**Keywords:** *Arcobacter* spp., *Aliarcobacter*, Ghana, Tanzania, Zoonotic transmission, Genome, Resistance, Virulence

## Abstract

**Background:**

*Aliarcobacter* spp. are emerging foodborne pathogens posing public health risks through zoonotic transmission in agricultural settings. Characterizing their phylogenetic relatedness, antimicrobial resistance (AMR), and virulence factors is critical for assessing their impact on food safety and health in Ghana and Tanzania.

**Methods:**

We collected 144 *Aliarcobacter* isolates (139 from farm animals and 5 from human clinical cases) and analyzed their genomes using molecular phylogenetics, antimicrobial resistance gene (ARG) profiling, and virulence gene identification.

**Results:**

Phylogenetic analysis confirmed *Aliarcobacter butzleri* as the predominant species with two distinct subclades exhibiting multiple drug resistance. Notably, we identified a second clade of *Aliarcobacter lanthieri* that may represent a novel species. Additionally, a potential new subspecies within *A. butzleri* was detected, supported by genomic and digital DNA:DNA hybridization (dDDH) analyses. A multiple antibiotic-resistant *Aliarcobacter cryaerophilus* isolate was found to harbor a resistance plasmid encoding five AMR genes. The pangenome reflected an open genome structure with a vast accessory gene pool related to resistance and virulence. ARGs conferring tetracycline, macrolide, and aminoglycoside resistance were widespread, with commercial farm isolates showing higher resistance burdens than those from smallholder farms. Virulence genes varied, illustrating broad genetic diversity and adaptive potential.

**Conclusions:**

This study highlights genomic plasticity and emerging resistance in *Aliarcobacter* spp. from human and animal sources in sub-Saharan Africa. The discovery of new clades and a resistant plasmid underscore the need for enhanced One Health surveillance to control the spread and impact of these pathogens on public health and food safety.

## Highlights

• *Aliarcobacter butzleri* predominates among *Arcobacter* isolates from Ghana and Tanzania
• Distinct *A. butzleri* subclades show multidrug resistance patterns.
• A potential new *A. lanthieri* clade/species and a potential new *A. butzleri* clade/subspecies was found.
• Resistance plasmid with five AMR genes was detected in *A. cryaerophilus*.
• Open pangenome shows large accessory pool for resistance and virulence genes.

## 1 Introduction

*Aliarcobacter* spp. are zoonotic foodborne pathogens linked to gastroenteritis, and bacteremia in humans, as well as enteritis, mastitis and abortion in animals [1], [2]. As of now, 36 species have been classified within the genus *Arcobacter* [List of Prokaryotic names with Standing in Nomenclature accessed November 2025] [3] and the following genera are recognized as heterotypic synonyms of *Arcobacter*: *Aliarcobacter* gen. Nov., *Pseudarcobacter* gen. Nov., *Halarcobacter* gen. Nov., *Malaciobacter* gen. Nov., and *Poseidonibacter* gen. Nov [4]. *Aliarcobacter* spp. are commonly present in the normal microbiota of warm-blooded animals, particularly in poultry and livestock [5], [6]. *Aliarcobacter butzleri*, *Aliarcobacter cryaerophilus*, and *Aliarcobacter skirrowii* are the three species most frequently isolated from human clinical specimens. Among these, *Aliarcobacter butzleri* is recognized as the primary cause of clinical infections [1], [7]. *A. butzleri* particularly affects older adults (>60 years) [8]. A French clinical cohort documented 1204 human *Aliarcobacter* infections confirming substantial disease burden in hospitalized patients [8]. Lithuanian poultry meat isolates (*n* = 27) exhibited high pathogenicity potential, with 100% carrying virulence genes *cad*F, *cia*B, *mvi*N, *pld*A, and *tly*A that facilitate intestinal adherence and invasion [9], [10]. German waterfowl strains from Thuringia and Brazilian clinical/environmental isolates further demonstrate *A. butzleri*'s genomic capacity for human infection, underscoring its underreported burden akin to related Campylobacteraceae [11]. Some *Aliarcobacter* infections can become invasive, potentially leading to severe complications such as sepsis, brain abscesses, lung infections, septic arthritis, and meningitis [1], [12], [13]. As *Aliarcobacter* is an emerging pathogen, its global prevalence and pathogenicity are still not sufficiently studied [4]. Limited research has been conducted on the epidemiology of *Aliarcobacter* in Africa [6], [14], [15]. However, a recent study from two sub-Saharan countries found that nearly half of the *Aliarcobacter* isolates from farms were multidrug-resistant [14]. This high rate of multidrug resistance is likely attributed to the indiscriminate use of antibiotics in animal husbandry [16]. A recent comparative genome-wide analysis of *A. butzleri* strains led to the detection of putative antimicrobial resistance genes and potential virulence factors contributing to the ability of the bacteria to adhere to and invade human intestinal cells [10], [17]. Poultry meat isolates exhibit high resistance, with 81.5% (22/27) of *A. butzleri* strains resistant to ≥1 antibiotic class and 55.5% displaying multidrug resistance (MDR), particularly to clindamycin (77.7%), florfenicol (62.9%), nalidixic acid (59.2%), and measurable macrolides (azithromycin 11.1%) [9]. Water-poultry strains from Thuringia, Germany showed phenotypic resistance to fluoroquinolones and tetracyclines, corroborated by genomic detection of ARGs [11]. Rarer species like *A. cibarius* and *A. thereius* carry resistance mutations (e.g., *gyr*A T86I), while systematic meta-analysis confirms *Arcobacter* prevalence averaging 30% along the poultry-centered food chain, highlighting the genus's role in disseminating clinically relevant resistance [6]. Horizontal gene transfer is proposed as the mechanism responsible for the presence of similar insertion sequences, which are implicated in mutations, observed in three different *Aliarcobacter* species isolated from poultry [18]. Genomic analysis of *Aliarcobacter* is becoming increasingly important as antimicrobial resistance and virulence genes are detected in both established and newly described potentially pathogenic species [1], [19]. The etiologic role of *A. butzleri* in human enteritis has been well established, along with its repertoire of virulence-associated genes (VAGs) and antimicrobial resistance genes (ARGs). In contrast, the roles of other species within the genus—such as *A. cryaerophilus*, *Aliarcobacter lanthieri*, and *Aliarcobacter vitoriensis*—in human disease remain disputable. Moreover, the specific functions of virulence factors and the mechanisms underlying antibiotic resistance in most *Aliarcobacter* spp. are still poorly understood. Systematic meta-analysis reveals *Aliarcobacter* prevalence averaging 30% (95% CI 24–34%) along the farm-to-retail continuum, peaking in poultry products, raw milk (25%), and environmental water (28.1%), with *A. butzleri* predominant [6]. Livestock serve as fecal reservoirs (cattle, sheep, pigs, poultry), enabling zoonotic spillover via contaminated food/water [20]. Lithuanian poultry meat and German waterfowl strains confirm *A. butzleri*'s ubiquity in poultry production systems [11], [17]. This poultry-centric transmission mirrors *Campylobacter* pathways, positioning *Aliarcobacter* as a One Health concern [6].

Overall, gaps in our knowledge of epidemiology, pathogenicity, antimicrobial resistance, virulence, and transmission routes hinder our ability to accurately assess their prevalence and the potential health risks they pose. To the best of our knowledge, only a few comprehensive comparative genomic analyses have been conducted on *Aliarcobacter*, all within Europe [17], [18] with just one from Africa [21]. Consequently, there remains a knowledge gap regarding the pathogenicity and epidemiology of this bacterium in the African context. Therefore, the present study aims to conduct a comparative genome analysis of *Aliarcobacter* genomes obtained from commercial and smallholder farms in two sub-Saharan African countries, Ghana and Tanzania. The objective is to characterize the genomes, determining their genetic diversity, pan-genomic structure, putative virulence factors, and resistance mechanisms.

## 2 Methods

### 2.1 *Aliarcobacter* isolates

A total of 144 *Aliarcobacter* isolates (Supplemental File 1), comprising 126 from Ghana and 18 from Tanzania, were included in this study. Of these, 97 were isolated from commercial farms, 42 from smallholder farms, 3 from children with diarrhea, and 2 from children without diarrhea (chicken: 70, swine: 38, quail: 10, duck: 9, human: 5, goat: 4, cow: 4,turkey: 2, sheep: 2). All strains were identified at the species level using Matrix-Assisted Laser Desorption/Ionization Time-of-Flight Mass Spectrometry (MALDI-TOF MS) using the VITEK® MS system (bioMérieux, Marcy-l'Étoile, France). In a previous study, we reported the prevalence and antimicrobial resistance trends of *Aliarcobacter* spp. isolated from commercial and smallholder farm animals, including chickens, swine, goats, quail, ducks, turkeys, and cattle, in Ghana and Tanzania [14], and the same samples were used in the current study. All 144 strains were stored at −80 °C using the Microbank™ system (Pro-Lab Diagnostics, Bromborough, UK). Before genomic DNA (gDNA) extraction, strains were cultured on Mueller-Hinton agar plates supplemented with 5% (*v*/v) sheep blood at 30 °C for 48 h under microaerophilic conditions generated by CampyGen (Oxoid, UK).

### 2.2 Ethical approval

The study was approved by the Committee on Human Research, Publications and Ethics, School of Medical Sciences, Kwame Nkrumah University of Science and Technology, Kumasi, Ghana (No. CHRPE/AP/674/19); by the Tanzania Medical Research Coordinating Committee (MRCC) hosted at the National Institute for Medical Research (NIMR/HQ/R.8a/Vol-IX/2909); and by the Ethikkommission der Ärztekammer Hamburg, Germany (No. PV5664). Participants were informed of the study's purpose and procedures, and written informed consent was obtained from each child's parent or guardian prior to enrollment.

### 2.3 Genome Sequencing

MasterPure™ Complete DNA and RNA Purification Kit was utilized for DNA extraction prior to whole-genome sequencing (WGS). *Aliarcobacter* cells from a 48 h culture plate were transferred into Eppendorf tubes containing 0.45% NaCl. After centrifugation and removal of the supernatant, the cell pellets were vortexed to dissociate tightly bound pellets formed due to auto-agglutination. Proteinase K was then added, followed by incubation at 65 °C. The samples were subsequently treated with RNase and cooled before adding MPC Protein Precipitation Reagent (LGC Biosearch Technology, Petaluma, California, USA) to remove debris. Isopropanol was used to precipitate DNA, which was washed with ethanol and air-dried before re-suspension in Tris-EDTA Buffer. DNA concentration and purity were assessed using Nanodrop spectrophotometric platform (ThermoFisher Scientific, USA) and Qubit fluorometric platform (Thermofisher Scientific, USA), respectively. WGS of all the *Aliarcobacter* isolates was performed between October and December 2022 at the Institute of Medical Microbiology and Hospital Hygiene, Medical Faculty, Otto von Guericke University Magdeburg, Germany. The Illumina MiSeq System (Illumina, California, USA) was used for sequencing, with library preparation conducted using the NEBNext® Ultra™ II FS DNA Library Prep Kit (Illumina, NEB #E7805S/L, #E6177S/L). Libraries were assessed for quality and loaded onto the MiSeq Reagent Kit v3 (Illumina, USA, MS-102-3003). The Illumina MiSeq System was configured to run 300 bp paired-end reads in FastQ generation mode. Barcoded libraries for Oxford Nanopore long-read sequencing were prepared using the Rapid Barcoding Kit 96 (SQK-RBK110.96) according to the manufacturer's instructions and sequenced on aR9.4.1 flow cell (FLO-MIN106) on the MinION platform (Oxford Nanopore technologies ltd., Oxford, United Kingdom). Illumina paired-end reads were preprocessed using fastp (https://github.com/OpenGene/fastp, v0.23.2) and filtlong (parameters: –min_length 1000 –keep_percent 95, https://github.com/rrwick/Filtlong, v0.2.1) [22].

### 2.4 Bioinformatics

#### 2.4.1 Genome assembly and annotation

Initial quality control was performed using FastQC v0.12.1 [23], followed by trimming and filtering of the reads with Trimmomatic [24] to ensure the removal of low-quality sequences. Genomes were assembled by Unicycler v0.5.0 [25] using the standard parameters for a hybrid assembly (4 threads, modus “normal”). Assembly quality was assessed with QUAST v5.2.0 with default parameters [quast.py assembly.fasta] [26]. Taxonomic assignment was performed using Mash v2.3 by sketching query genomes with mash sketch -o sample sample.fna and comparing them against a taxonomically annotated Mash reference database using mash taxscreen [27]. The *Aliarcobacter* genomes were annotated using the online NCBI Prokaryotic Genome Annotation Pipeline (PGAP) version 6.9 accessed as indicated in each individual GenBank entry [28].

#### 2.4.2 Phylogenetic analysis

Core genomes were calculated using Roary version 3.13 [29] utilizing all gnomes included in this study. Roary was run with the parameters -r -z -e -i 90, using a 90% sequence identity threshold for gene clustering. Maximum-likelihood trees were constructed using RAxML v8.2.12 [30]. The GTR (General Time Reversible) model was applied for nucleotide substitution, while the CAT model approximated rate heterogeneity across sites. Sequences with ≥ 90% identity were clustered, and only genes present in at least 95% of the sequences were considered in the analysis. Phylogenetic analysis was performed on the nexus file generated from the RAxML output, and the tree was visualized as a circular phylogenetic tree using iTOL v6.5.1 [31]. The tree was annotated with genomic features, sample host and source, and geographical origin to explore the evolutionary relationships between the genomes.

#### 2.4.3 Antimicrobial resistance and virulence gene profiling

To assess the ARGs of the *Aliarcobacter* isolates, we conducted an initial ResFinder 4.7.2 search [32] [80% Threshold for ID and 60% for Minimum length] for each bacterial genome. The identified hits were added to our curated database/FASTA file of ARGs. Similarly, a database/FASTA file was compiled for virulence-associated factor genes (VAGs) of *Aliarcobacter* and *Campylobacter* described in the literature (see Supplementary Tables 1 and 2 for legends). Subsequently, a BLAST search was conducted against a database of ARGs and VAGs. The genomes were screened by mapping sequences to known resistance and virulence gene references, applying a minimum identity threshold of ≥90% to ensure high-confidence gene hits. Heatmaps were generated to visualize the level of identity, calculated as the number of identical nucleotides divided by the length of the input sequence, using R version 4.4.1 [33].

#### 2.4.4 Pangenome analysis

Gene prediction and annotation were performed with Prokka v1.14.6 [34]. Gene clustering was carried out using Roary v3.13.0 [29] with a 90% identity threshold to group homologous proteins. The gene_presence_absence.Rtab output from Roary was used as the presence-absence matrix; for the pan-and-core-genome analyses a 95% inclusion threshold was applied. To generate pan-and-core-genome-plots, the sample order was randomized 100,000 times for *A. lanthieri* and 1000 times for *A. butzleri*, and for each permutation the number of genes in the core genome or pangenome among the first N samples was recorded.

## 3 Results

### 3.1 Genome sequencing and assembly

We have sequenced a total of 144 genomes from four different *Aliarcobacter* species, specifically 132/144 (91.7%) *A. butzleri*, 9/144 (6.3%) *A. lanthieri*, 2/144 (1.4%) *A. cryaerophilus* genomovar *pseudocryaerophilus*, and 1/144 (0.7%) *A. vitoriensis*. In this study, we aimed to close all non-*A. butzleri* genomes using both long and short read sequencing, achieving success with all but one isolate of *A. lanthieri*. Additionally, we closed 11 *A. butzleri* genomes through long and short read sequencing, primarily due to the potential presence of resistance plasmids. Assembly statistics were available for 139 of the 144 assembled *Aliarcobacter* genomes. The vast majority (138/139, 99.3%) exhibited >99% completeness, and only two genomes had estimated contamination exceeding 5%. The mean quality score, defined as completeness minus five times contamination, was 99.1% (range: 57.6–99.9%). Assembly sizes ranged from 1.8 to 2.5 Mb (mean: 2.2 Mb). Contig counts per genome varied from 1 to 832 (median: 33), and N50 values ranged from 3027 bp to 2,317,166 bp; most assemblies were highly contiguous, with 130/139 (93.5%) showing N50 > 100 kb. Notably, five genomes (3.6%) were fully circularized (single contig), and 13 assemblies (9.4%) contained fewer than 15 contigs.

### 3.2 Phylogenetic analysis of *Aliarcobacter* isolates

Phylogenetic analysis of the *Aliarcobacter* isolates revealed strong species-specific clustering. *A. butzleri* formed the predominant superclade, which can be further subdivided into two clades. The nine *A. lanthieri* isolates clustered into two distinct clades, the two *A. cryaerophilus* isolates belonged to the genomovar *pseudocryaerophilus*, and a single isolate was identified as *A. vitoriensis*.

Isolates from various host species, including chicken (*n* = 70), swine (*n* = 38), quail (*n* = 10), ducks (*n* = 9), human (*n* = 5), goat (*n* = 4), cow (*n* = 4), sheep (*n* = 2), and turkey (*n* = 2), were dispersed throughout the phylogenetic tree without host-associated clustering. While the majority of isolates were from commercial farms (*n* = 97), those from smallholder farms (*n* = 42) and humans (*n* = 5) were distributed throughout the same major clades. Similarly, isolates from Ghana (126/144, 87.5%) and Tanzania (18/144, 12.5%) were interspersed across the major clades, with no evidence of geographic segregation. The majority of internal nodes (73/128, 57.0%) had bootstrap values ≥70%, supporting the robustness of the tree topology. However, 55 internal nodes (43.0%), primarily within the *A. butzleri* clade, had bootstrap values <70%, suggesting some uncertainty in specific evolutionary relationships (Fig. 1).

**Fig. 1:**
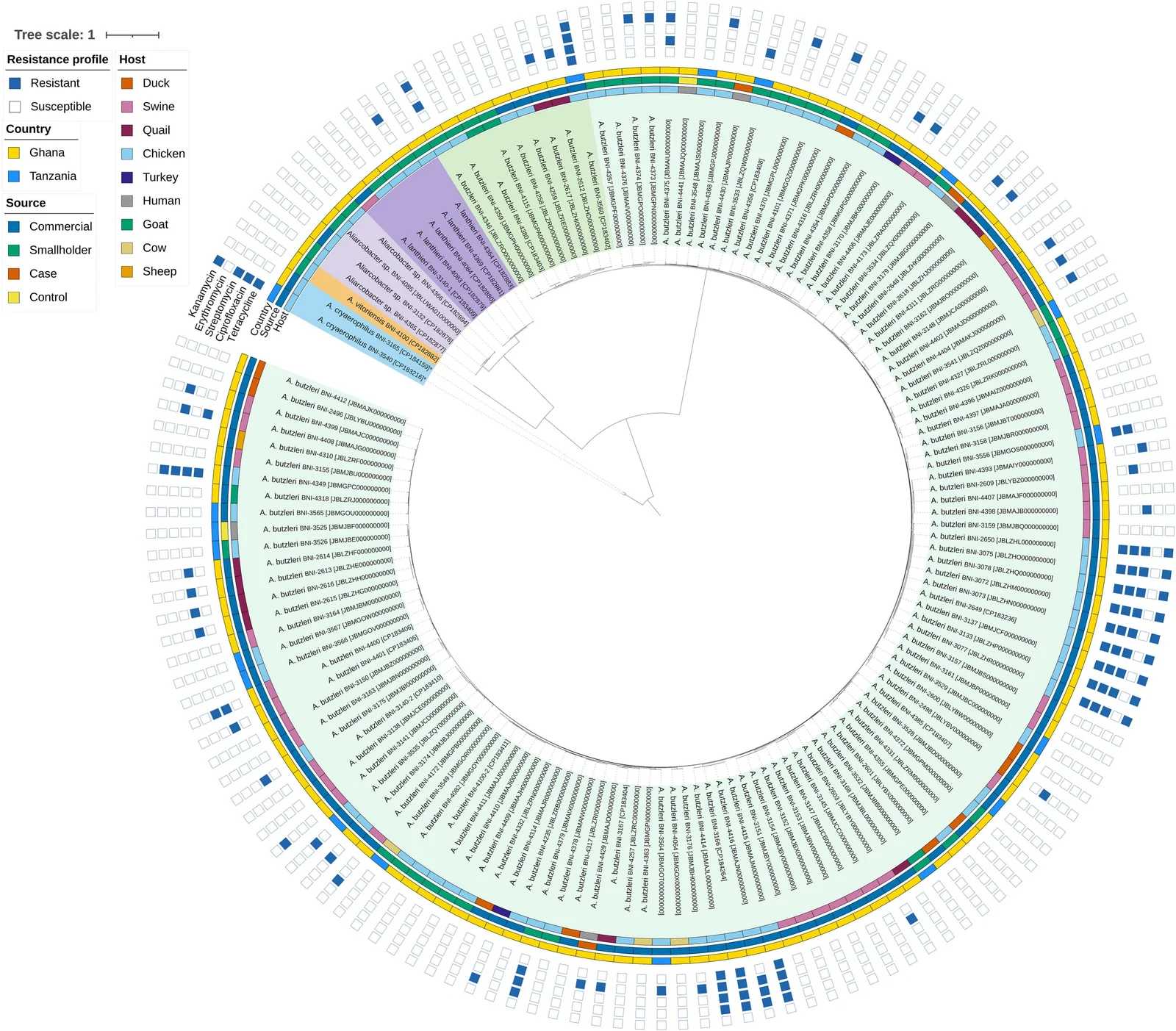
Phylogenetic tree of 144 *Aliarcobacter* isolates from Ghana and Tanzania, comprising 132 *A. butzleri*, 9 *A. lanthieri*, 2 *A. cryaerophilus* (gv. *pseudocryaerophilus*), and 1 *A. vitoriensis* isolates. The Maximum-likelihood phylogenetic tree was inferred with RAxML v8.2.12 based on the core genome alignment generated by Roary v3.13 using a 90% sequence identity threshold (−i 90) and a 95% core gene presence cutoff. The GTR model was used for nucleotide substitution, with the CAT approximation applied to account for rate heterogeneity across sites. GenBank accession numbers for each strain are shown in square brackets; bootstrap values ≥60% (1000 replicates) are indicated in blue at the nodes. Branch lengths are proportional to evolutionary distance (scale bar shown). Country, source, host, phenotypic resistance and clade of each isolate are indicated by the accompanying colour code. (For interpretation of the references to colour in this figure legend, the reader is referred to the web version of this article.)

### 3.3 *Aliarcobacter butzleri* isolates

The *A. butzleri* pan- and core-genome was analyzed using 132 genomes sequenced in this study. Based on these 132 genomes, the pangenome comprises 13,689 protein-coding genes (Fig. 2A). Of these, 1426 genes (10.4%) belong to the core genome (present in ≥99% of genomes, Fig. 2B) and 193 genes (1.4%) to the soft-core genome (present in ≥95% and < 99% of genomes). The accessory genome contains 12,070 genes (88.2%), including 716 genes (5.2%) in the shell genome (present in ≥15% and < 95% of genomes) and 11,354 genes (82.9%) in the cloud genome (present in <15% of genomes). Within the cloud genome, 5264 genes (38.5% of the pangenome; 46.4% of the cloud genome) are singletons (present in only one genome).

**Fig. 2:**
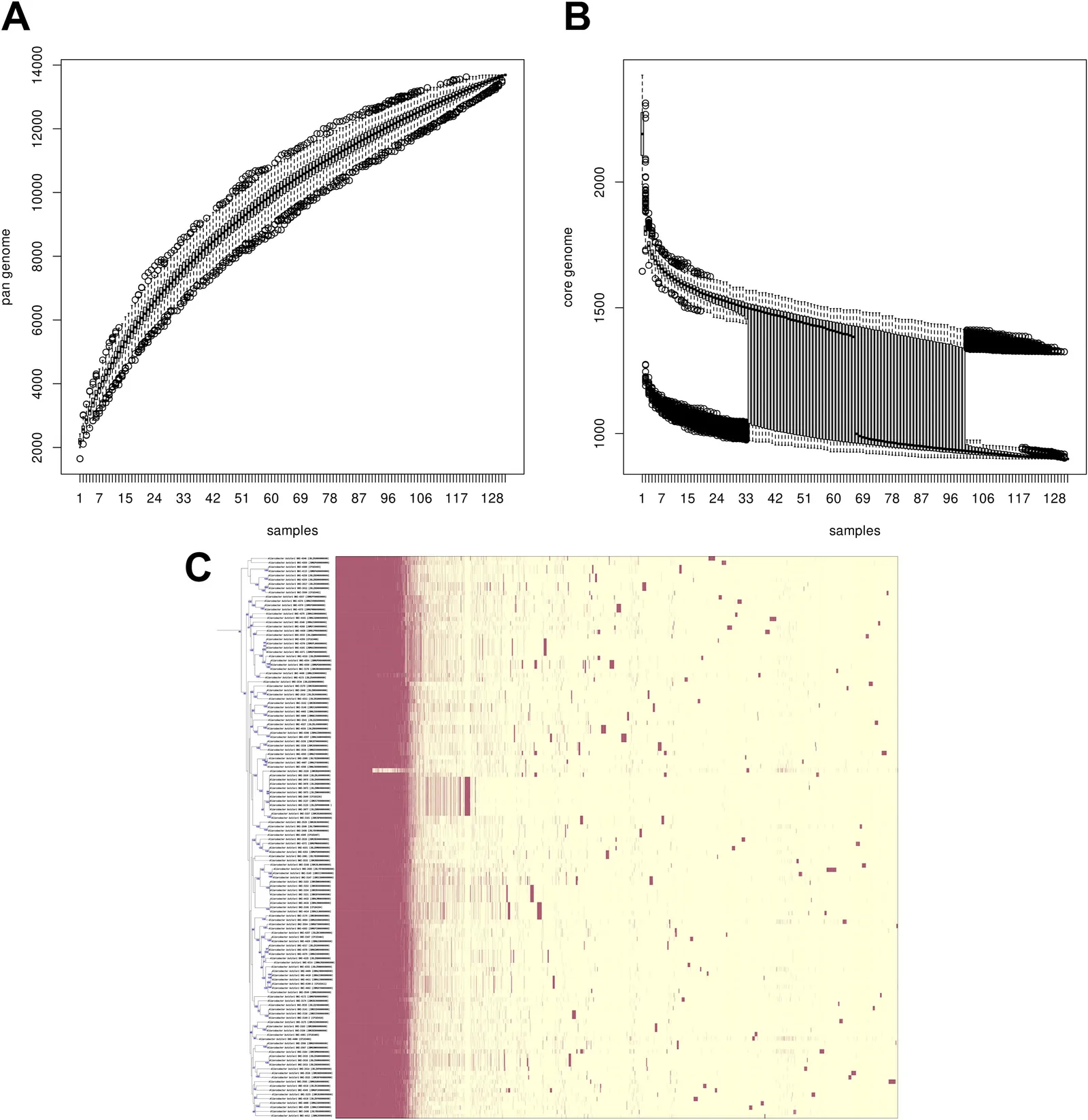
Pangenome analysis of 132 *A. butzleri* strains isolated from smallholder and commercial farms in Ghana and Tanzania: **A.** The diagram shows the cumulative number of genes in the pangenome as additional genomes are added. **B.** The diagram shows the successive decreasing number of conserved genes in the pangenome as additional genomes are added. **C.** The hierarchical tree on the left shows the *A. butzleri* subtree from Fig. 1 and defines the order of isolates in the gene presence–absence matrix on the right; therefore, each row of the matrix corresponds to a branch in the tree. Each column represents an orthologous gene cluster. Claret lines indicate gene presence, and pale-yellow lines indicate gene absence. Specific gene clusters in subclade a, which contains nine isolates, are labeled 1–9. (For interpretation of the references to colour in this figure legend, the reader is referred to the web version of this article.)

Additionally to the phylogenic tree, we calculated the average nucleotide identity (ANI) values for the 132 genomes and presented them as a matrix (Fig. 3) in the order of the obtained phylogeny. Both analyses initially indicate a split of the examined *A. butzleri* cohort into two clades. The numerically smaller clade consists of nine isolates: BNI-4346, BNI-4359, BNI-4380, BNI-4115, BNI-4258, BNI-4259, BNI-2617, BNI-2612, and BNI-3560. Within this clade, ANI values range from 97.3 to 100.0 (mean 98.1), whereas ANI values between these isolates and the other *A. butzleri* isolates range from 94.9 to 97.1 (mean 97.0). A TYGS analysis of the nine isolates produced dDDH (d4) values between 77.0% and 78.3%, which—when compared with the remaining 123 genomes—supports the existence of a distinct subspecies cluster. Furthermore, the subclade consisting of isolates BNI-4357 to BNI-4173 was notable in contrast to the group BNI-3534 to BNI-4412. Additionally, four clonal clusters were identified—BNI-4356 to BNI-4372, BNI-2650 to BNI-3077, BNI-3151 to BNI-3154, and the cluster comprising BNI-4415 and BNI-4414—and the subclade BNI-3176 to BNI-3549 was also distinguishable. The frequency distribution histogram (Fig. 2B) revealed also a two-clade subdivision within the *A. butzleri* cohort.

**Fig. 3:**
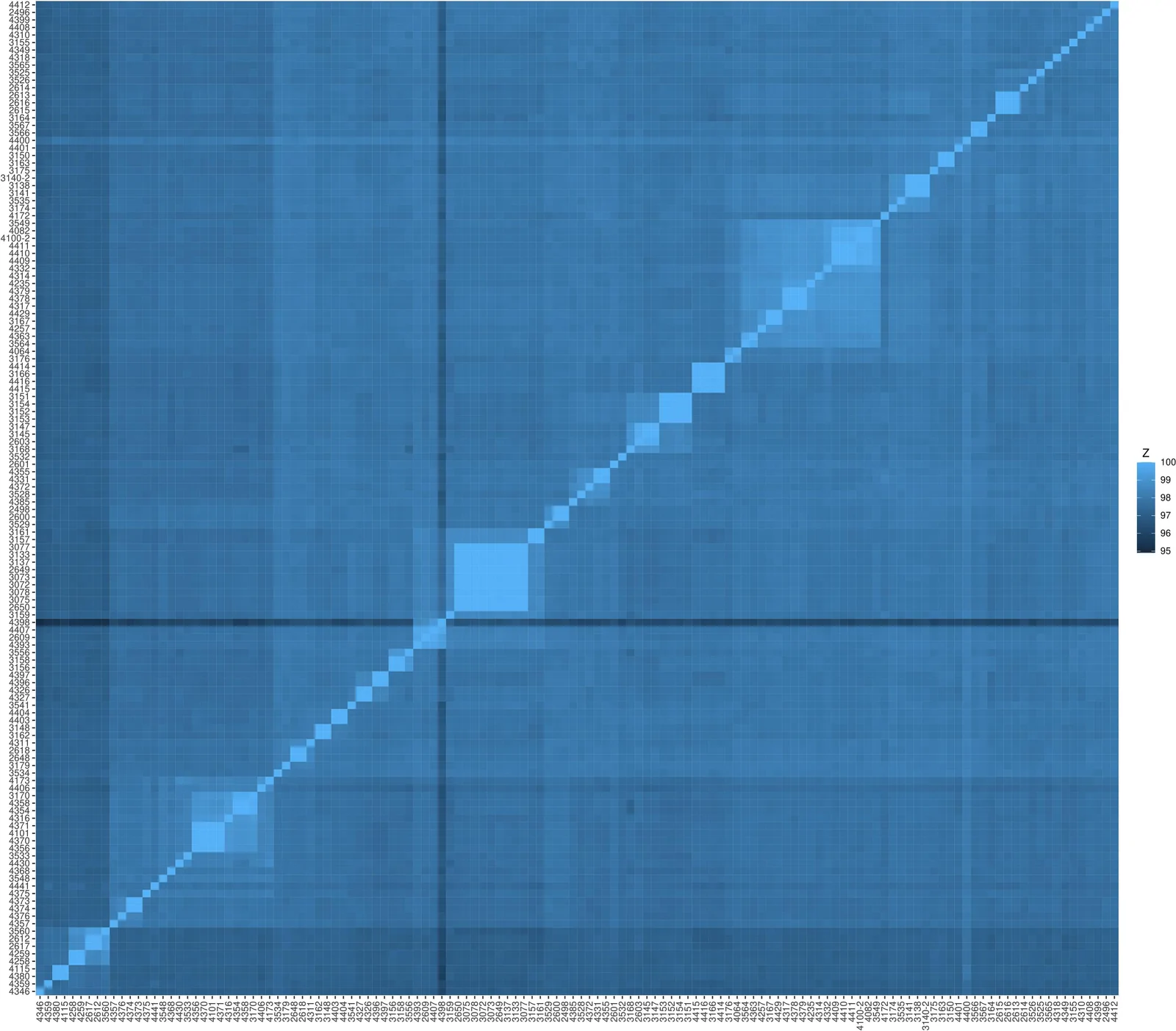
Heatmap of average nucleotide identity (ANI) among 132 *A. butzleri* genomes. The genomes are ordered according to the phylogeny in Fig. 1. Blue intensity corresponds to ANI percentage. ANI values of approximately 95–96% or higher indicate the same species, and values ≥98% indicate the same subspecies. In the analysis, a small clade of nine isolates — BNI-4346, BNI-4359, BNI-4380, BNI-4115, BNI-4258, BNI-4259, BNI-2617, BNI-2612, and BNI-3560 — shows ANI values between these isolates and the other *A. butzleri* genomes ranging from 94.91 to 97.71 (mean 97.00), supporting the existence of a distinct subspecies cluster. (For interpretation of the references to colour in this figure legend, the reader is referred to the web version of this article.)

Using the gene presence–absence matrix, several distinct clusters within the accessory genome were identified (Fig. 2C). Due to the large number of accessory gene clusters, we focused on the eight major clusters from the smaller second clade, which comprises nine isolates. These clusters include genes related to motility, nutrient acquisition, antibiotic resistance, DNA modification, and mobile genetic elements. A key feature across many clusters is the presence of iron acquisition systems, such as ferric anguibactin and ferripyoverdine receptors, highlighting the importance of iron uptake for this organism's survival and virulence. Some clusters encode proteins involved in bacterial defense and interaction, including colicin receptors and flagellar components, which enable motility and competitive advantages in microbial communities. Others harbor genes for enzymes that confer resistance to antibiotics, such as beta-lactamases and tetracycline resistance proteins, emphasizing the organism's capability to withstand antimicrobial agents. DNA modification systems, especially restriction-modification enzymes and methyltransferases, were prevalent and serve dual roles in protecting the bacterium from extraneous DNA and facilitating gene regulation. Several clusters encode prophage integrases, invertases, and recombinases, which are classical markers of mobile genetic elements. These enzymes mediate the integration, excision, and rearrangement of DNA segments, promoting horizontal gene transfer and genome plasticity. In particular, one cluster exhibits a strong signature of mobile genetic elements with transposases, serine recombinases, integrases, and genes linked to resistance and DNA modification. This supports the hypothesis that these gene clusters contribute to the genome's ability to acquire and disseminate advantageous traits, such as resistance and virulence factors. Moreover, clusters involved in sulfur and nucleotide metabolism contribute to metabolic versatility, enabling the bacterium to adapt to various environmental conditions. Other clusters encode enzymes related to redox processes and metal resistance, which are critical for coping with oxidative stress and toxic compounds in different niches. Overall, the accessory genome of *A. butzleri* clade 2 reflects a complex interplay between core survival functions and mobile genetic elements shaping genomic diversity. This structure allows the organism to maintain key pathogenic traits and acquire new capabilities through horizontal gene transfer, which drives its evolution and adaptation. The clustering of such diverse gene functions in discrete genomic loci also suggests coordinated regulation and mobility, contributing to the bacterial lifestyle in various environments and possibly enhancing its ability to cause infection and resist antimicrobial treatments.

### 3.4 *Aliarcobacter lanthieri* and related isolates

We successfully cultured and sequenced a total of 9 isolates that were initially identified by MALDI-TOF MS (VITEK® MS) as *A. lanthieri*, all of which were obtained from Ghana. The host animal was chicken, with 2 isolates coming from commercial farms and 7 from smallholder farms. For 8 of these isolates, we were able to assemble a fully closed circular chromosome. Additionally, one isolate, BNI-4366, contained an extrachromosomal element, a plasmid (CP182895) measuring 170,477 bp, which encodes a total of three CRISPR arrays. The phylogenetic analysis (Fig. 1) of the nine genomes clearly reveals a division into two clades, consisting of four and five isolates, respectively. To further validate this bifurcation, we conducted an additional phylogenetic analysis (Fig. 4C) incorporating the *A. lanthieri* genomes available in the NCBI GenBank, expanding the dataset to a total of 16 genomes (including *Aliarcobacter* sp. CECT 9188 as well as two assemblies of *A. lanthieri* LMG 28516^T^). This analysis also distinctly shows a separation into two clades, with the four-member group now including two additional genomes, these of *A. lanthieri* R-75363 [GCF_016756415] and *Aliarcobacter* sp. CECT 9188 [GCF_003316735]. In the next step, we calculated the ANI values for the 16 genomes and presented them as a matrix (Fig. 5). Here, these six genomes are clearly distinguishable. They exhibit ANI values ranging from 95.5% to 96.0% when compared to the clade of *A. lanthieri* isolates, which includes the reference strain LMG 28516^T^. Within their own group, however, they show ANI values ranging from 98.5% to 99.7%.

**Fig. 4:**
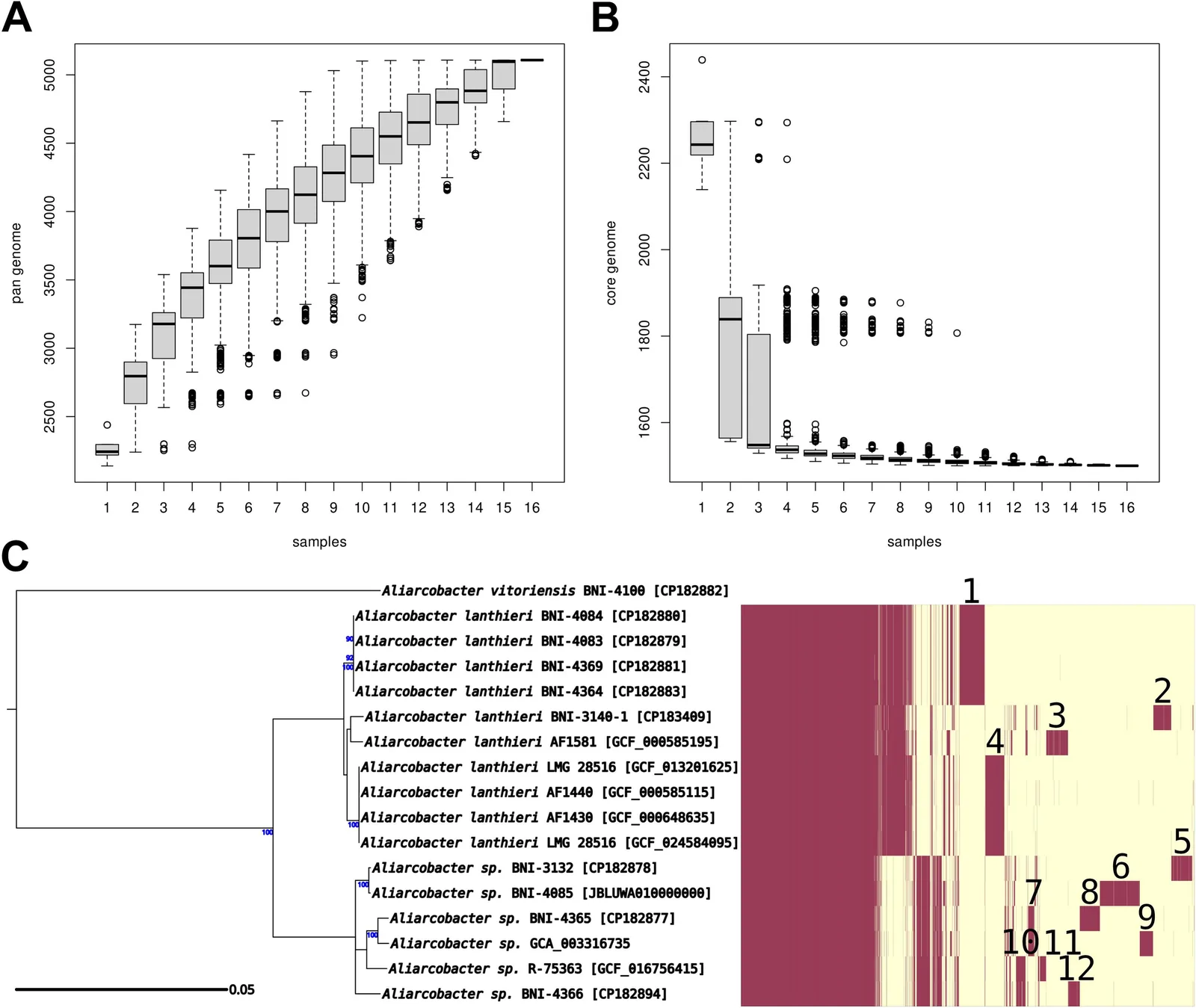
Pangenome analysis of 10 *A. lantheri* and 6 related *Aliarcobacter* sp. isolates including 9 isolates from smallholder and commercial farms in Ghana and Tanzania as well 6 Genomes obtained from NCBI-Genbank: **A.** The diagram shows the cumulative number of genes in the pangenome as additional genomes are added. **B.** The diagram shows the successive decreasing number of conserved genes in the pangenome as additional genomes are added. **C.** The hierarchical tree on the left was inferred with RAxML v8.2.12 using a 90% sequence identity threshold, a 90% presence cutoff, and the GTR model with a CAT approximation for rate heterogeneity. GenBank accession numbers for each strain are shown in square brackets; bootstrap values ≥60% (1000 replicates) are indicated in blue at the nodes. Branch lengths are proportional to evolutionary distance (scale bar shown). The tree defines the order of isolates in the gene presence–absence matrix on the right; therefore, each row of the matrix corresponds to a branch in the tree. Each column represents an orthologous gene cluster. Claret lines indicate gene presence, and pale-yellow lines indicate gene absence. Specific gene clusters are labeled 1–12. (For interpretation of the references to colour in this figure legend, the reader is referred to the web version of this article.)

**Fig. 5:**
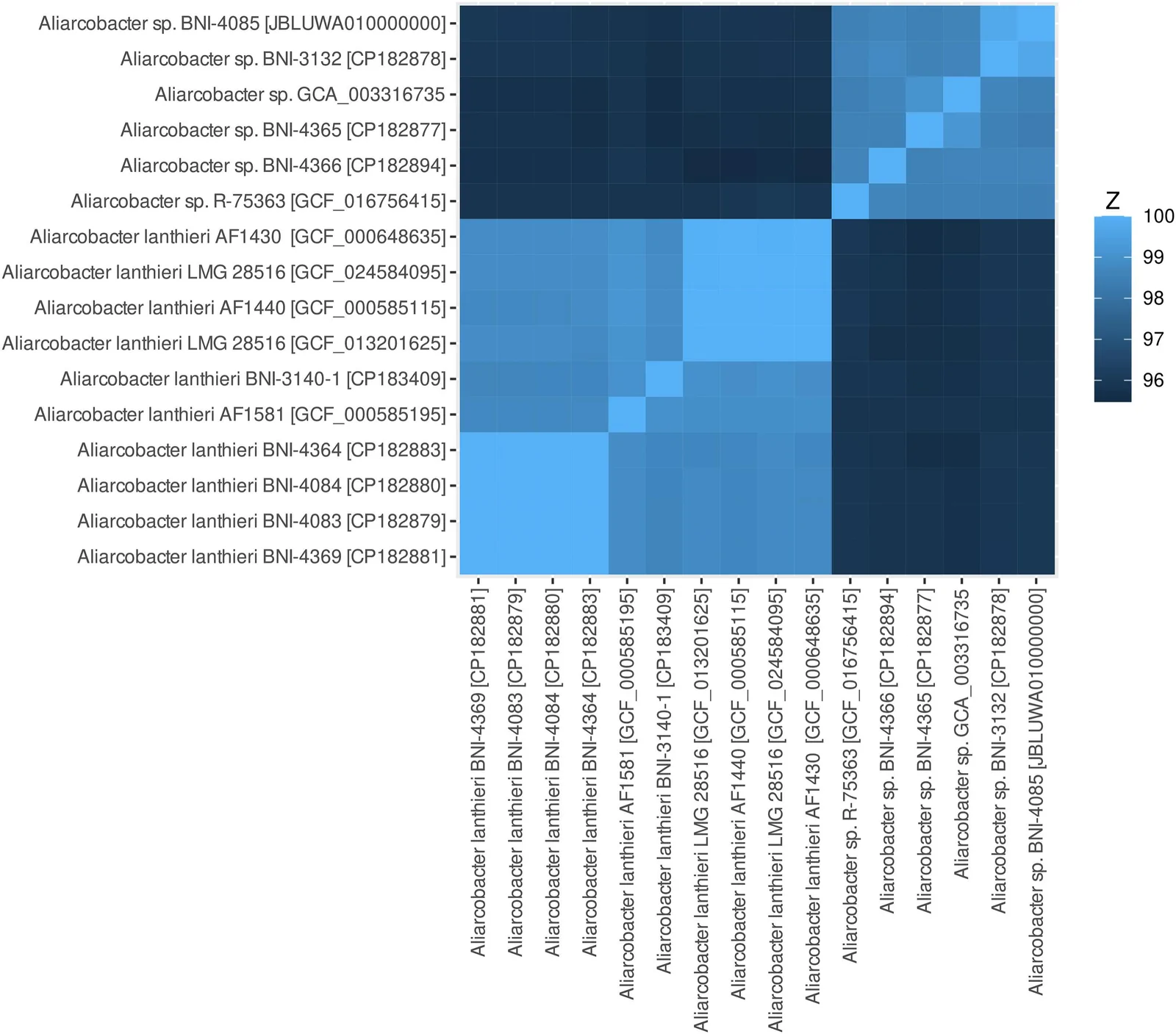
Heatmap of average nucleotide identity (ANI) among 16 *Aliarcobacter* genomes: 10 *A. lanthieri* isolates (including the type strain *A. lanthieri* LMG 28516 T) and 6 related *Aliarcobacter* sp. isolates. The dataset comprises nine isolates from our study, collected from smallholder and commercial farms in Ghana and Tanzania, plus six genomes retrieved from NCBI GenBank. Blue intensity corresponds to ANI percentage. ANI values of approximately 95–96% or higher indicate the same species, and values ≥98% indicate the same subspecies. (For interpretation of the references to colour in this figure legend, the reader is referred to the web version of this article.)

An *A. lanthieri* pan- and core-genome analysis was performed on 16 genomes, including those available from NCBI GenBank; these also encompass genomes from two clades and *A. lanthieri*–related isolates that may represent a separate species. Using these 16 genomes, the *A. lanthieri* pangenome comprised 5108 protein-coding genes (Fig. 4A). Of these genes, 1500 (29.4%) constituted the core genome (present in ≥99% of genomes), and 0 (0%) were assigned to the soft-core genome (95% ≤ presence <99%). The accessory genome comprised 3608 genes (70.6%), of which 1533 (30.0% of the pangenome) belonged to the shell genome (15% ≤ presence <95%) and 2075 (40.6% of the pangenome) to the cloud genome (presence <15%). Within the cloud genome, 1750 genes were singletons (*N* = 1), representing 34.3% of the pangenome and 84.3% of the cloud genome. The frequency-distribution boxplots (Fig. 4B) further highlights the two-clade structure within the *A. lanthieri* cohort. The gene presence–absence matrix revealed 12 distinct clusters within the accessory genome (Fig. 4C). The twelve clusters of the accessory *A. lantheri* genome mainly encode proteins for iron acquisition, surface glycosylation, toxin–antitoxin systems, DNA modification, and transport/resistance mechanisms. Eight clusters (1, 2, 3, 4, 6, 8, 9, 12) contain numerous siderophore receptor genes (*fat*A, *fhu*E, pyoverdine receptor genes), highlighting an emphasis on iron uptake under limiting conditions, likely essential for virulence and survival. Clusters 1, 3 and 4 harbor legionaminic and bacillosaminic acid biosynthesis genes, contributing to LOS and capsule decoration, thereby supporting immune evasion and host adaptation. Clusters 5 and 6 involve competence genes (*com*M) and CRISPR elements (Cas1, Cas12a), indicating roles in DNA uptake, recombination, and phage defense. Other clusters (7, 11, 12) encode restriction/modification systems (*eco*RI, *dde*I, *hha*I) and recombinases (*xer*D, *int*A), suggesting active genome plasticity and epigenetic control. Clusters 8 and 9 contain genes encoding efflux components (*mac*B, *cus*C, *mex*AB-*opr*M) conferring antibiotic and metal resistance, whereas 11 and 12 include Type IV secretion systems (*vir*B complex) linked to DNA conjugation and virulence. Some clusters also encode toxin-antitoxin modules (*hip*A, *rel*E/*lso*A) enabling stress adaptation and persistence. Overall, three major functions emerge: iron and metal homeostasis, surface and host interaction mechanisms, immune defense, resistance, and genome mobility. Shared functional features of these gene clusters lie in metal utilization, recombination, and stress protection, while differences reflect a shift between iron acquisition, glycan biosynthesis, and resistance systems. Together, these patterns indicate that accessory gene clusters play an important role in adaptation to hosts and ecological niches.

### 3.5 *Aliarcobacter cryaerophilus* genomovar *pseudocryaerophilus*

As part of our study, we successfully cultured two *A. cryaerophilus* isolates, both originating from commercially raised chickens. One isolate is from Ghana (BNI-3165), and the other is from Tanzania (BNI-3540). Both belong to the *pseudocryaerophilus* genomovar, which we confirmed through a TYGS analysis that included published genomovar type strains. For both isolates, we were able to obtain fully closed genomes using a combination of Oxford Nanopore and Illumina sequencing. The chromosome of BNI-3540 (CP183216) was 1,938,861 bp in size and contained 2158 genes, of which 2083 were protein-coding, 49 were for tRNAs, five each for 5S, 16S, and 23S rRNAs, two for ncRNAs, and nine that were pseudogenes. The genome included one CRISPR array. The chromosome of BNI-3165 (CP184159) differs in that it has a size of 1,985,209 bp and encoded for 2312 genes, of which 2068 were protein-coding, 48 were tRNA genes, and 179 were pseudogenes. Furthermore, the genome of BNI-3540 contains an extrachromosomal element, a plasmid measuring 150,772 bp, which is particularly notable for encoding five antimicrobial resistance genes: *tet*(M), *aac*(6′)/*aph*(2″), *ant*(6)-Ia, *aph*(3′)-III, and *tet*(Y).

### 3.6 Aliarcobacter vitoriensis

Finally, we successfully cultured the only *A. vitoriensis* isolate (BNI-4100) from a chicken on a Ghanaian smallholder farm in our study. Through parallel long-read and short-read sequencing, we obtained a complete circular and closed genome for this isolate (CP182882). The genome was 2,352,861 bp in size and contained 2335 genes, of which 2266 were protein-coding, 49 were for tRNAs, five each for 5S, 16S, and 23S rRNAs, two for ncRNAs, and three that were pseudogenes. The genome included a CRISPR array and does not contain any extrachromosomal elements. Among the specific resistance determinants, it harbors the bla_OXA−464_ class D beta-lactamase gene.

### 3.7 Antimicrobial resistance gene profiles across genomes

Among the 144 genomes analyzed, the most prevalent antibiotic resistance genes (ARGs, Fig. 6) were *mac*A and *mac*B2 (each 100%, 144/144), *mac*B1 (99.3%, 143/144), and *tol*C (99.3%, 143/144). These genes are all associated with macrolide resistance; however, *erm*B — a gene often implicated in high-level macrolide resistance — was not detected in any of the isolates. The *ade*F gene, which encodes a subunit of a periplasmic adaptor of a multidrug efflux RND transporter and whose role in antimicrobial resistance is unclear, was detected in 100% of the genomes. The genes encoding the subunits of the bile acid transporter—*cme*A, *cme*B, *cme*C, and *cme*R—which can also confer quinolone resistance, were ubiquitous. The C254T mutation in the *gyr*A gene's quinolone resistance-determining region (QRDR), which confers quinolone resistance in *Aliarcobacter* spp., was detected in 25.6% (34/133) of isolates, was absent in 99/133, and could not be evaluated in 11/144 genomes because the contig containing *gyr*A was missing. The presence of the β-lactamase genes *bla*_VEB_ and *bla*_OXA-464_ was investigated. *bla*_VEB_ was not detected in any genome. In contrast, *bla*_OXA-464_ was identified in 76.4% (110/144) of isolates with high sequence identity and coverage, and in an additional 15.3% (22/144) with reduced identity/coverage, which may indicate the presence of a pseudogene. The gene was absent in 8.3% (12/144) of isolates. Notably, all nine *A. lanthieri* isolates (including the 4 *A. lanthieri* related isolates) and one of two *A. cryaerophilus* gv. *pseudocryaerophilus* isolates were *bla*_OXA-464_–negative, meaning only 2 of 132 *A. butzleri* isolates lacked *bla*_OXA-464_. As noted, above the genome of *A. cryaerophilus* gv. *pseudocryaerophilus* BNI-3540 contains a resistance plasmid encoding five antibiotic resistance genes: *tet*(M), *aac*(6′)/*aph*(2″), *ant*(6)-Ia, *aph*(3′)-IIIa, and *tet*(Y); this exact combination has been observed only in this isolate. While *tet*(M), *aac*(6′)/*aph*(2″), *ant*(6)-Ia, and *aph*(3′)-IIIa were found exclusively in BNI-3540, *tet*(Y) was also detected in two additional *A. butzleri* isolates. The genome of *A. butzleri* BNI-3166 contained a further resistance plasmid, p152-BNI-3166, carrying *tet*L, *aad*D, *ble*O, *msr*D, and *mef*A. These antibiotic resistance genes (ARGs), on closely related plasmids, were also found in the four *A. butzleri* isolates BNI-4414, BNI-4415, and BNI-4416, that form a clonal group, and in the unrelated isolate BNI-4349. The three genes *tet*L, *aad*D, and *ble*O were additionally detected on a 65-kb plasmid (p65) in a clonal group comprising nine isolates: BNI-2649, BNI-2650, BNI-3072, BNI-3073, BNI-3075, BNI-3077, BNI-3078, BNI-3133, and BNI-3137. This p65 plasmid was completely sequenced from strain BNI-2649. The ARG *tet*L was also detected in one other isolate. The chromosomally encoded streptomycin resistance gene *str* was present in 11.8% (17/144) of isolates, predominantly in the two clonal groups mentioned above. The aminoglycoside resistance gene *aad*A2 was detected in 1.4% (2/144) of isolates. The resistance genes *tet*A, *ere*B, *cat*, and *optr*A, previously reported in *Aliarcobacter*, were not detected in any isolate from the test cohort. The ARG burden per genome was similar between isolates from Ghana and Tanzania (5.1 vs. 5.2; rate ratio [RR] = 1.0, 95% CI 0.8–1.3). In contrast, isolates from commercial farms exhibited a higher ARG burden than those from smallholder farms (5.6 vs. 4.0; RR = 1.4, 95% CI 1.2–1.7).

**Fig. 6:**
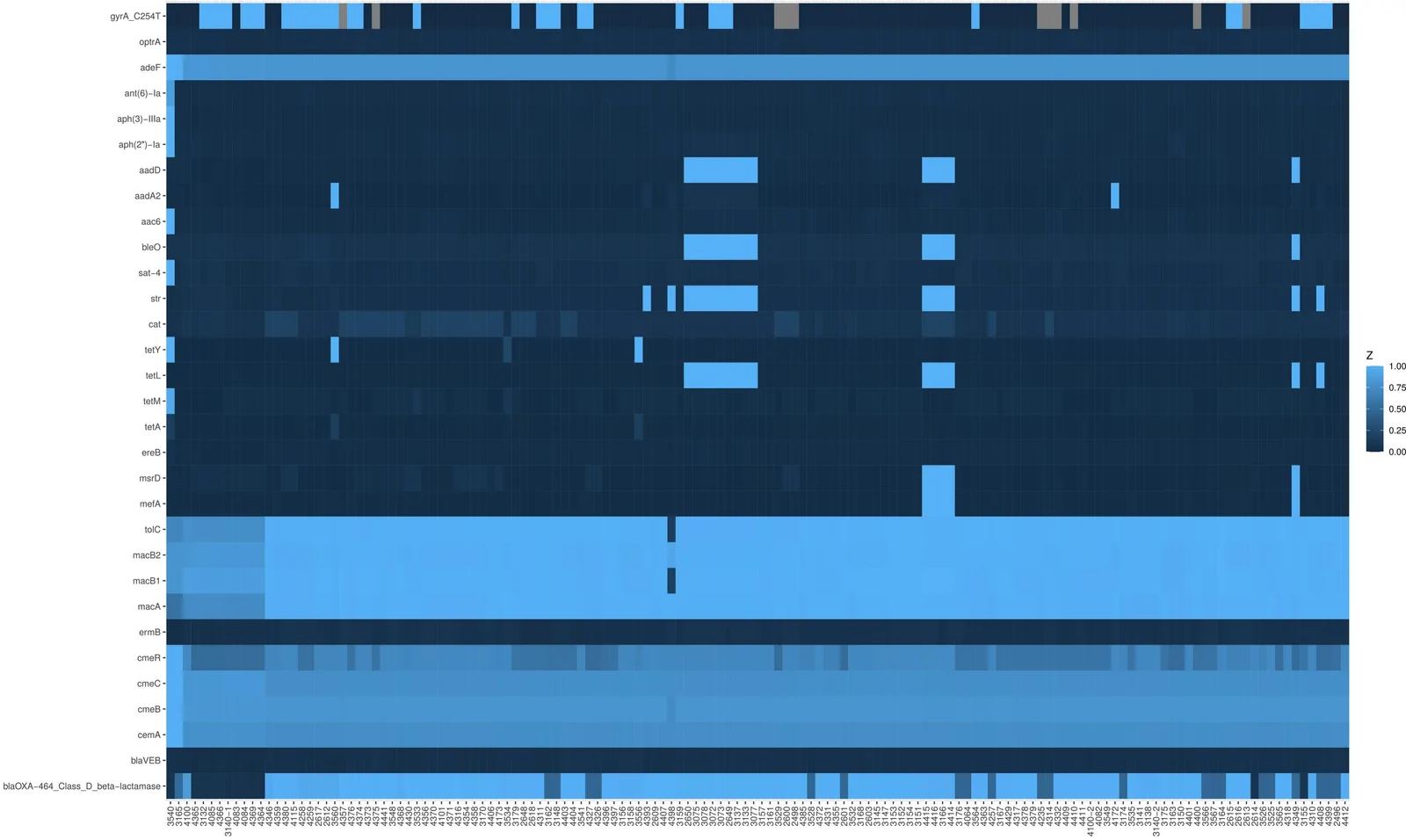
Heatmap indicating the presence of *Aliarcobacter* antimicrobial resistance genes. Each column represents one *Aliarcobacter* isolate. The *Aliarcobacter* genomic sequences are organized in phylogenetic order based on the dendrogram presented in Fig. 1. The lines represent 31 antimicrobial resistance genes or antimicrobial susceptibility associated point mutation (legend see Supplementary Table 1). The intensity of the blue colour corresponds to the % identity from the BLAST analysis. (For interpretation of the references to colour in this figure legend, the reader is referred to the web version of this article.)

Regarding the correlation between phenotypic resistance reported in our previous publication [14] and the presence of detected resistance genes, the following associations should be highlighted. Phenotypic tetracycline resistance was observed in 17 of 144 (11.8%) isolates. Among these, 14 (82.4%; 14/17) harbored *tet*Y. The gene *tet*L was detected in the other three isolates (17.6%; 3/17), and one of these isolates carried both *tet*L and *tet*M. One further isolate was phenotypically susceptible to tetracycline and *tet*Y-positive, potentially indicating a defective *tet*Y gene.

Of the 144 isolates, 16 were erythromycin-resistant (11.1%; 16/144). In five of these isolates (31.3%; 5/16), an association with both the *msr*D gene (encoding the ABC-F–type ribosomal protection protein MsrD) and the *mef*A gene (encoding the macrolide efflux MFS transporter MefA) was detected; in the remaining 11 isolates (68.8%; 11/16), erythromycin resistance was not associated with any known macrolide resistance gene. A streptomycin-resistant phenotype was observed in 16.7% (24/144) of the isolates. Among these, 70.8% (17/24) were associated with detection of *str* (aminoglycoside adenylyltransferase family protein), 8.3% (2/24) with *aad*A2 (AadA family aminoglycoside 3”-*O*-nucleotidyltransferase), and 4.2% (1/24) with the *sat*-4 gene (streptothricin *N*-acetyltransferase Sat4). In the remaining 16.7% (4/24) of isolates, no association with any of the tested resistance genes was found, suggesting that other mechanisms confer streptomycin resistance. Using agar diffusion kanamycin resistance was observed in 6.9% (10/144) of the isolates, this was associated in 90% (9/10) with the genes *ble*O (bleomycin binding protein) and *aac*6 and in one isolate (10%; 1/10) with the four genes *aac*6, *aph*(2″) − Ia, *aph*(3) − IIIa, and *ant*(6) − Ia. However, 5 isolates tested positive for *ble*O and *aac*6 were phenotypically kanamycin susceptible. Of the isolates, 31.9% (46/144) were resistant to ciprofloxacin. Among these resistant isolates, 23.9% (11/46) carried the *gyr*A C254T amino acid substitution, while *gyr*A was not detected in the assembled contigs of 8.7% (4/46). By comparison, the *gyr*A C254T substitution was detected in 23.5% (23/98) of ciprofloxacin-susceptible isolates, and *gyr*A was not detected in the contigs of 7.1% (7/98) of susceptible isolates. Given the similar prevalence of the C254T substitution in both resistant and susceptible groups, a causal relationship between this amino acid substitution and ciprofloxacin/quinolone resistance is questionable. Overall, *A. cryaerophilus* isolate BNI-3540 and two clonal groups of *A. butzleri* (BNI-2650, BNI-3075, BNI-3078, BNI-3072, BNI-3073, BNI-2649, BNI-3137, BNI-3133, and BNI-3077; and BNI-4415, BNI-4416, BNI-3166, and BNI-4414) exhibit phenotypic multidrug resistance and harbor multiple antibiotic resistance genes in their genomes.

### 3.8 Diversity of virulence genes

Across the 144 *Aliarcobacter* genomes, we surveyed 89 distinct putative virulence-associated genes (VAGs, Fig. 7). Some of these genes are representative of specific virulence mechanisms and therefore not practical to display individually. The VAGs were grouped into 13 functional categories: (1) Type IV secretion system (T4SS) — associated with genetic flexibility e.g. in *C. fetus*, (2) reactive oxygen species (ROS) inactivation, (3) capsular/O-antigen synthesis, (4) lipooligosaccharide (LOS) synthesis and modification, (5) anaerobic respiration, (6) amino acid metabolism, (7) hemolysis and iron transport, (8) membrane integrity and modification, (9) RND-family efflux pumps, (10) flagellar motility, (11) pseudaminic acid biosynthesis/flagellar glycosylation, (12) *N*-linked glycosylation, and (13) adhesion and invasion factors. A heatmap shows gene presence or absence and the percentage of sequence identity, indicated by graduated shades of blue (Fig. 7). Our analysis revealed distinct distribution patterns of virulence-associated genes across different *Aliarcobacter* species and clades. (1.) A complete, functional Type IV secretion system (T4SS) was not detected in any of the isolates; only the singular *vir*B6 gene was detected in 16.0% (23/144) of the genomes. Among the VAGs involved in (2.) reactive oxygen species (ROS) inactivation, the *sod* (superoxide dismutase) and *per*R (peroxide repressor) genes were detected in 100% of isolates. The *kat*E gene, which encodes a catalase, was found in *A. cryaerophilus*, *A. vitoriensis*, *A. lanthieri*, and related isolates, but was absent from all *A. butzleri isolates*. In *Aliarcobacter* spp., a canonical *kps* gene cluster for capsule polysaccharide synthesis like that of *Campylobacter jejuni* is absent; instead, (3.) genes implicated in *O*-antigen and capsule polysaccharide biosynthesis are scattered throughout the genome. The heatmap reveals five distinct *A. butzleri* subclades — isolates BNI-3548 to BNI-4173, BNI-4327 to BNI-4397, BNI-4257 to BNI-3549, BNI-3174 to BNI-3140-2, BNI-2616 to BNI-3525 — in which the gene set *wbp*I, *tvi*B, *wbp*G, *wbp*E, *wbp*D, and *wbp*B is largely intact. The same pattern is also observed in one *A. lanthieri*–related isolate, BNI-4366. As in *Campylobacter*, *Aliarcobacter* species possess a shortened (4.) lipooligosaccharide (LOS) compared with the lipopolysaccharide (LPS) of other Gram-negative bacteria. The glycosyltransferase gene products *gmd*-1, *wec*C, *wbj*D, *gtr*B, and *gal*E are essential for LOS synthesis in *Aliarcobacter*. By contrast, the genes *wbt*I, *wbt*A, *gac*A, *rfb*C, *uge*, *ugd*, *neu*C2, and *neu*B2 encode factors involved only in LOS modification. Although each of these genes was detected in varying numbers of isolates, none of the genomes examined contained the complete set of essential glycosyltransferase genes, indicating that none of the livestock isolates studied are likely capable of synthesizing a complete LOS. Among the factors we examined for (5.) anaerobic respiration, the periplasmic nitrate reductase gene *nap*A was ubiquitous, whereas the three subunits of the dimethylsulfoxide (DMSO) reductase were not detected in any of the genomes. The two genes, (6.) *pan*D and *his*F, which can be regarded as VAGs in the context of amino acid metabolism, were also found to be ubiquitous. Within (7.) the category “hemolysis and iron transport”, the hemolysin-encoding gene *tly*A was ubiquitous. By contrast, the hemolysin-activation protein gene *hec*B was primarily present in two *A. butzleri* subclades (BNI-4356 to BNI-4173 and BNI-3564 to BNI-3549). Additionally, two *A. lanthieri* isolates (BNI-3140-1 and BNI-4366) were *hec*B-positive. In these two *A. lanthieri* isolates and in the *A. butzleri* subclade BNI-4356 to BNI-4173, the filamentous hemagglutinin gene *hec*A was also detected. Furthermore, *Aliarcobacter* genomes contain a range of iron transport proteins that can functionally complement or substitute for one another; some of these proteins cannot be fully distinguished due to high sequence identity. Notable aspects of the distribution of iron transport genes include the absence of the iron export gene *fie*F in *A. lanthieri* (and related) isolates, and the presence of *iro*E in 100% of *A. vitoriensis* and *A. lanthieri* isolates and in 55.3% (73/132) of the *A. butzleri* isolates. The *irg*A gene was detected in only 22.2% (2/9) of *A. lanthieri* isolates and was absent from all other *Aliarcobacter* species. The TonB-dependent iron transporter gene *fcu*A1 was absent from the two *A. cryaerophilus* isolates but detectable in 88.9% (8/9) of *A. lanthieri* isolates, in 83.3% (110/132) of *A. butzleri* isolates, and in the single *A. vitoriensis* isolate. The related gene *fcu*A2 was absent from *A. cryaerophilus* and *A. lanthieri* isolates but was present in 81.1% (107/132) of *A. butzleri* isolates and in *A. vitoriensis* BNI-4100. While *cir*1 was ubiquitous in *A. butzleri*, clade-specific distribution was observed among *A. lanthieri* isolates with *cir*1 being present only in clade 2. In contrast, *cir*2 was detected in all nine *A. lanthieri* isolates (i.e., in both clades) but in only 21.2% (28/132) of *A. butzleri* isolates. Finally, the ubiquitous transcriptional regulator *tbd*RI, *tbd*RII was detected in 3 of 4 *A. lanthieri* isolates and in 64.4% (85/132) of *A. butzleri* isolates. Among the genes examined in category 8, membrane integrity and modification, *fab*Z, *acp*XL, and *eno* were ubiquitous across the isolate cohort. *lpl*A-like was absent from *A. cryaerophilus* but ubiquitous in isolates of the other three species. Gene *bpl*B showed a distinct distribution: it was detected in a single *A. cryaerophilus* isolate, in one clade 2 *A. lanthieri* isolate, and in 47.7% (63/132) of *A. butzleri* isolates. The genes encoding subunits A and B of the (9.) RND efflux pump AcrAB-TolC always co-occurred or were consistently absent. While all isolates of species other than *A. butzleri* were *acr*A/B-positive, both genes were detected in 31.8% (42/132) of *A. butzleri* isolates, with a predominance in subclades BNI-4258–BNI-3560, BNI-4357–BNI-4173 and BNI-3168–BNI-3151. The (10.) cluster, consisting of nine genes associated with flagellar motility, was detected in all isolates. For (11.) pseudaminic acid biosynthesis/flagellar glycosylation, the gene products of *pse*A, *pse*B, *pse*F, and *pse*I are considered essential. All four genes were detected together in 44.4% (4/9) of *A. lanthieri* isolates and in 28.0% (37/132) of *A. butzleri* isolates. Of these *A. butzleri* isolates, 5.4% (2/37) additionally carried *ptm*B, a gene regarded as nonessential. Regarding (12.) *N*-linked glycosylation, the four genes examined — *pgl*C, *pgl*D, *pgl*E, and *pgl*F — and their gene products are considered essential. Only the isolate *A. cryaerophilus* BNI-3540 was tested positive for all four genes, indicating a functional *N*-linked glycosylation pathway for protein modification. Finally, the five genes associated with (13.) adhesion and invasion — *cia*B, *htr*A, *pld*A, *cj1349*-like, and *cad*F-like — were ubiquitous in the studied isolates.

**Fig. 7:**
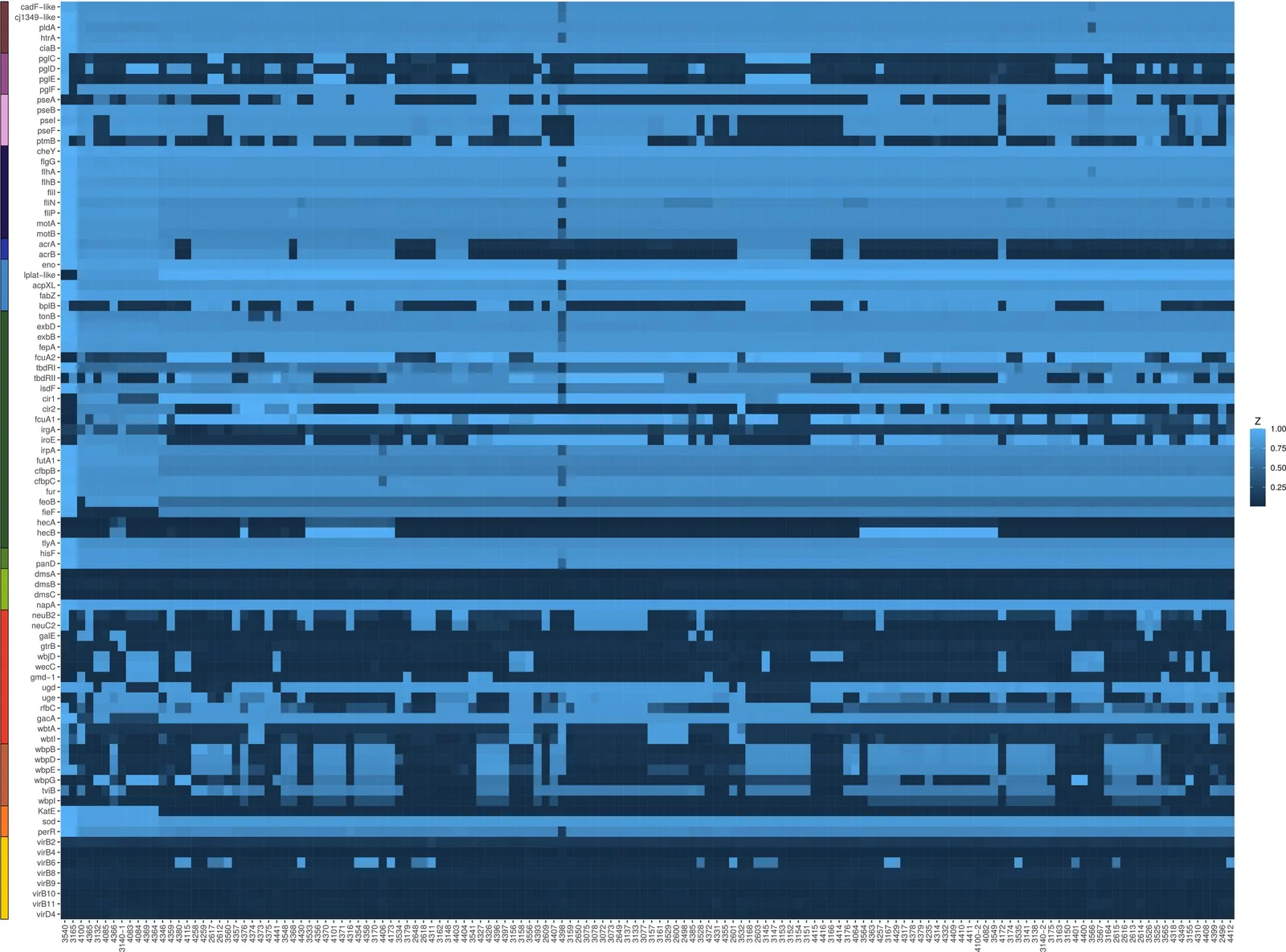
Heatmap showing the presence of *Aliarcobacter* virulence-associated genes. Each column corresponds to a single *Aliarcobacter* isolate. Genomes are arranged in phylogenetic order according to the dendrogram in Fig. 1. Rows represent 89 virulence- and resistance-associated genes (legend in Supplementary Table 2). Blue colour intensity indicates percent identity as determined by BLAST analysis. The colour code for the 13 functional categories is: (1 - yellow) Type IV secretion system (T4SS) (2 - orange) ROS inactivation, (3 - brown) capsular/O-antigen synthesis, (4 - red) LOS synthesis and modification, (5 - light green) anaerobic respiration, (6 - hunter green) amino acid metabolism, (7 - dark green) hemolysis and iron transport, (8 - light blue) membrane integrity and modification, (9 - royal blue) RND-family efflux pumps, (10 - dark blue) flagellar motility, (11 - lavender) pseudaminic acid biosynthesis/flagellar glycosylation, (12 - violet) N-linked glycosylation, and (13 - mulberry) adhesion and invasion factors. (For interpretation of the references to colour in this figure legend, the reader is referred to the web version of this article.)

## 4 Discussion

Our genomic characterization of 144 *Aliarcobacter* spp. isolates from commercial and smallholder farms as well as children in Ghana and Tanzania provides valuable insights into the genomic diversity, AMRs and VAGs, across different host species, sample sources, and geographic regions of sub-Sharan Africa. Despite strong species-level clustering, the absence of distinct *A. butzleri* sub-clades based on host species, sample source, or geographic origin suggests that *Aliarcobacter* spp. exhibit high genomic plasticity, enabling them to modify and adapt their genomes to diverse environmental conditions, host species, and ecological niches. This adaptability is consistent with previous findings showing that *A. butzleri* has a large open pan-genome containing numerous unique genes, which enhance its ability to colonize different environments and hosts through a broad array of virulence factors and AMR mechanisms [35]. Similarly, comparative genomic analyses of Arcobacteraceae have identified specific genes associated with environmental and host adaptation, further supporting the evolutionary flexibility of this genus [19]. The interspersion of isolates from diverse hosts—ranging from poultry and livestock to humans—throughout the phylogenetic tree further suggests weak host specificity and the potential for cross-host transmission among *Aliarcobacter* spp. [19], [20]. These findings align with previous studies proposing *Aliarcobacter* as an opportunistic, free-living pathogen with a broad host range, capable of adapting to various ecological niches [1], [36], [37]. Additionally, the lack of phylogeographic segregation between isolates from Ghana and Tanzania indicates ongoing AMR gene flow and widespread dissemination of *Aliarcobacter* strains across regions, likely facilitated by factors such as trade, animal movement, and shared environmental reservoirs [11], [20], [38], [39], [40], [41].

The robustness of our phylogenetic tree is supported by strong bootstrap values (≥70%) in 57.0% of internal nodes, reinforcing confidence in the overall topology. However, the lower bootstrap support (<70%) in 43.0% of internal nodes—particularly within the dominant *A. butzleri* clade—suggests the presence of unresolved evolutionary relationships. This could be due to high recombination rates, horizontal gene transfer, or rapid genomic diversification, all of which can complicate the reconstruction of accurate phylogenies [10], [42], [43], [44]. Similar challenges in phylogenetic resolution due to horizontal gene transfer and recombination have been reported in other bacterial species, including *Campylobacter* [45]. For instance, *C. jejuni* lineages have been found to separate into two groups: those that specialize in particular host species and those that can infect a broad range of hosts [46], highlighting how host factors influence genomic diversity. Similarly, studies on the biodiversity, ecology, and evolution of reptile-associated Epsilonproteobacteria, including *Aliarcobacter*, show that these species exhibit a broad host range and significant genetic diversity, underscoring the role of host ecology in shaping their evolution and adaptation [20], [47]. These findings point to the need for further whole-genome-based analyses, including comparative genomics and pan-genome studies, to resolve strain-level variations and better understand the mechanisms driving *Aliarcobacter* evolution.

Our genomic analyses revealed distinct new clades within the *Aliarcobacter* isolates that extend the current understanding of species and subspecies boundaries. The subtree of our phylogenetic analysis that contains isolates/genomes initially identified by MALDI-TOF MS as *A. lanthieri* splits into two distinct clades. Pairwise average nucleotide identity (ANI) values between representatives of these clades range from 95% to 96%, suggesting that the clade comprising four isolates may represent a novel bacterial species besides *A. lanthieri*. Inclusion of the genomes *Aliarcobacter* sp. CECT 9188 and R-75363 in the phylogenetic analysis showed that both belong likewise to this second clade. *Aliarcobacter* sp. CECT 9188 is part of a group of *Aliarcobacter* isolates recovered from wastewater and river water samples collected around Vitoria-Gasteiz in northern Spain. For these isolates the species name “(*Ali*-)*Arcobacter hispanicus*” has been proposed, although it has not yet been validly published [48]. In addition to surface water, we now report chicken as another reservoir for this potentially novel *Aliarcobacter* species (i.e., the second *A. lanthieri* clade). A further publication describes isolate R-75363, recovered from an immunocompetent patient with persistent abdominal bloating and cramps without diarrhea or fever and with no reported travel history or animal contacts; this isolate also clusters within the same clade [49]. This singular clinical case provides preliminary evidence for human clinical relevance and potential enteropathogenicity of isolates from this clade/species. This finding aligns with recent studies highlighting complex genomic heterogeneity within *Aliarcobacter* spp., suggesting that taxonomic refinements are warranted as more genomes become available.

Similarly, we identified a distinct clade within *A. butzleri* that shows genomic signatures indicative of a potential new subspecies. These findings underscore the genomic plasticity of *Aliarcobacter* and highlight the importance of whole-genome analyses for accurate epidemiological and taxonomic assessments.

The ARG profiles across the analyzed *Aliarcobacter* genomes revealed a higher frequency of macrolide resistance genes, likely due to selective pressure from frequent macrolide use in clinical and agricultural settings [50], [51]. Macrolide resistance in *Aliarcobacter* is of particular concern, as these antibiotics are commonly used to treat infections caused by related Campylobacteraceae species, including *Campylobacter jejuni* [52], [53]. The agricultural use of macrolides as growth promoters and veterinary therapeutics may contribute to the selection and persistence of resistant *Aliarcobacter* strains, facilitating their spread between animals and humans [16], [54], [55]. Additionally, the β-lactam resistance gene *bla*_OXA-464_ was found in three-fourths of the *Aliarcobacter* genomes, conferring resistance to β-lactams, including carbapenems, which are critical for treating multidrug-resistant infections [56]. Intrinsic *bla*_OXA_-like genes in *Aliarcobacter* contribute to β-lactam resistance, with potential for environmental persistence as seen in other Epsilonproteobacteria [10], [42]. Comparison between isolates from commercial and smallholder farms revealed a higher ARG burden in commercial farms, likely due to intensive antibiotic use and close confinement of animals, which contribute to AMR accumulation and gene transfer [57], [58]. The detection of a highly diverse ARG profile in a single *Aliarcobacter* genome from a Tanzanian commercial farm underscores the potential of *Aliarcobacter* spp. as reservoirs of multidrug resistance [14], posing a risk of transmission to humans through the food chain or direct contact. Additionally, several resistance genes associated with tetracycline and aminoglycosides were identified in the genomes of the analyzed *Aliarcobacter* isolates. Specifically, the detection of tetracycline resistance genes in approximately one-tenth of the isolates, confirmed previous reports of tetracycline resistance mechanisms in *Aliarcobacter* spp. [21], [35]. The detection of specific glycopeptide and aminoglycoside resistance genes, in a subset of isolates, indicated potential resistance to this classes of antibiotics [21]. These specific ARGs underscore the capacity of *Aliarcobacter* spp. to acquire and disseminate resistance determinants, posing challenges for antimicrobial therapy and control.

Our study identified multiple antibiotic-resistant subclades of *A. butzleri*, emphasizing the public health implications of resistance dissemination within this species. Two *A. butzleri* subclades exhibited multidrug-resistant phenotypes with associated resistance genes including tetracycline, aminoglycoside and macrolide resistance determinants, signifying clonal expansion of resistant strains in both commercial and smallholder farms. Of particular concern, an *A. cryaerophilus* isolate was found to harbor a resistance plasmid encoding five antimicrobial resistance genes, a rare but critical mechanism for horizontal gene transfer of resistance traits in *Aliarcobacter*. This plasmid-mediated resistance highlights the ongoing evolution and adaptation of *Aliarcobacter* to antibiotic pressures and may represent an emerging threat requiring surveillance and containment strategies.

The striking diversity of virulence genes among *Aliarcobacter* underlines its pathogenic potential and ability to adapt to a wide range of hosts and environments [35]. The widespread presence of *eno* and *lplat* − like genes in over 90% of genomes underscores their fundamental role in adhesion and membrane stability, potentially facilitating host colonization and persistence [59], [60]. The high prevalence of iron acquisition genes, suggests that iron sequestration is a key survival strategy, enabling *Aliarcobacter* to thrive in nutrient-limited environments such as the host intestine [10], [61]. The detection of more than two-thirds of the pangenome's virulence genes within a single genome highlights the striking genetic heterogeneity of *Aliarcobacter* and suggests that different strains may employ distinct virulence strategies to establish infection [62]. This variability raises critical public health concerns, as certain strains may possess an enhanced ability to colonize hosts, evade immune responses, or persist in the food chain.

Compared with other recent genomics-based epidemiological studies on *Aliarcobacter*, it should be noted that differences in study design limit direct comparability with the data presented here. First, our own pilot study from Ghana examined 48 *A. butzleri* isolates from poultry meat using MLST-based phylogeny, antimicrobial susceptibility testing, and PCR screening for ARGs. Only two multidrug-resistant isolates were further characterized by whole-genome sequencing [21]. Second, researchers at the Friedrich-Loeffler-Institut Jena sequenced and characterized *Aliarcobacter* isolates from water poultry farms in Thuringia, Germany, including 45 *A. butzleri*, 27 *A. cryaerophilus*, two *Aliarcobacter cibarius*, and two *Aliarcobacter thereius* isolates [11], [63], [64]. Finally, the study by Uljanovas and colleagues, comprising 40 *A. butzleri* isolates mostly from poultry meat and including genome and pangenome analyses, has a study design most similar to our own [10]. All four studies have deposited their genomic data in publicly accessible databases (NCBI/ENA), so the number of available *Aliarcobacter* genomes is fortunately increasing. A comparison is most pertinent for the dominant *Aliarcobacter* species, *A. butzleri*. Phylogenetic analyses across all studies reveal a high degree of heterogeneity among sequence types recovered from diverse sources. None of the studies delineate a specific outbreak strain or a distinct pathovar [10], [64]. In contrast, our study identified a second clade of nine isolates that, based on ANI values, may represent an *A. butzleri* subspecies and could be characteristic of Ghana or of Africa more broadly.

Comparing antibiotic resistance across studies is complicated because our study used internally established Ecoff values to assess susceptibility [21], while the other studies applied EUCAST breakpoints for *Campylobacter* and *Enterobacterales* on *Aliarcobacter* isolates [10], [64]. In our two African studies, quinolone resistance was relatively high at 31.3% and 25.6%, compared with much lower rates of 4.4% in Thuringia (Germany) and 5.0% in Lithuania. Quinolone resistance was predominantly attributable to amino acid substitutions in the quinolone resistance–determining region (QRDR) [10], [21], [64].

The pattern is reversed for tetracyclines. In our African isolates, tetracycline resistance was 11.8% and 12.5%, associated mainly with *tet*L and *tet*Y genes. In contrast, tetracycline resistance was substantially higher in Europe — 35.0% in Lithuania and 44.4% in Thuringia — where the underlying molecular mechanisms are very heterogeneous and in some cases remain unidentified. A similar contrast is seen for macrolides. The two Ghanaian studies showed macrolide resistance of 10.4% and 11.1%, linked to *erm*B, *msr*D, and *mef*A. By comparison, macrolide resistance in Lithuania and Thuringia was markedly higher at 55.0% and 71.1%, respectively. In Europe, the genetic determinants of macrolide resistance are again highly heterogeneous and partly unidentifiable. Determining ampicillin resistance is particularly difficult because our data do not allow a robust Ecoff to be defined [21]. Elevated MICs or reduced inhibition zone diameters for ampicillin were associated across studies with the presence of *bla*3/*bla*_OXA-464_ genes. Detection rates of these genes or highly similar variants were relatively high in Ghana (72.9% and 76.4%) and Lithuania (87.5%), but comparatively low in water poultry from Thuringia (37.8%). In our study, an additional 15.3% of *bla* genes showed lower sequence identity to *bla*_OXA-464_ and likely represent *bla*_OXA-464_–like and *bla*_OXA-15_–like (*bla*2) variants [10], [64]. The observed geographic differences in resistance phenotypes and ARG prevalence likely reflect different patterns of antibiotic use. Quinolone use appears to be more prevalent in animal husbandry in parts of Africa, whereas tetracyclines seem to be more commonly used in Europe. Resistance to Aminoglycoside, which are used in African livestock production, played a lesser role in the European datasets [16], [65], [66]. The reasons for the high macrolide resistance observed in Europe remain speculative and may be related to macrolide use in human medicine. Comparing virulence-associated genes (VAGs) across studies is challenging because different sets of VAGs were surveyed and different methodologies were applied. In all studies, a large proportion of VAGs—particularly those involved in motility and chemotaxis—were found to be ubiquitous [10], [64]. No complete, functional Type IV secretion system (T4SS) was detected, although individual genes encoding T4SS components were identified in some isolates. Diverse iron acquisition and transport systems were detected across genomes and may functionally substitute for one another. Adhesion and invasion factor genes (*cia*B, *htr*A, *pld*A, *cj1349*-like, and *cad*F-like) were ubiquitous in *A. butzleri* isolates from all studies. The gene complement for lipid A and core oligosaccharide biosynthesis is likewise ubiquitous, whereas genes for further LOS synthesis show marked variability. No or only rudimentary O-antigen biosynthesis is present, resulting in truncated lipooligosaccharides (LOS). Overall, these zoonotic *A. butzleri* strains can be considered potential human and animal pathogens with theoretically variable virulence that can only be inferred genomically in colonizing strains lacking clinical manifestation. Moreover, it should be noted that, in addition to *A. butzleri*, these studies have increased the number of isolates and genomes available for the so-called “rare” *Aliarcobacter* species. In our study we identified *A. cryaerophilis*, *A. lanthieri*, *A. vitoriensis*, and “*A. hispanicus*,” and isolates of *A. cryaerophilis*, *A. cibarius*, and *A. thereius* from waterfowl in Thuringia were characterized and sequenced [11], [63].

Despite conducting a comprehensive genomic analysis of *Aliarcobacter* spp. across commercial and smallholder farms and children in Ghana and Tanzania, several limitations must be noted. The sampling was geographically constrained to specific communities in Ghana and Tanzania, potentially limiting the generalizability of findings to other regions of Sub-Saharan Africa. Additionally, the study did not fully consider the influence of environmental factors, such as farming practices and local antimicrobial policies, on *Aliarcobacter* strain diversity and resistance profiles. A further limitation is the small number of human-derived genomes (only 5 out of 144), which were insufficient for meaningful subgroup analysis of human-associated strains. This limited the ability to draw robust conclusions regarding the specific impact of *Aliarcobacter* on human health in these regions. The overarching goal of this work was to characterize *Aliarcobacter* transmission dynamics comprehensively using a One Health approach. A major limitation of the current study, particularly with respect to providing a concise overview of infection routes, is that samples were predominantly obtained from food and farm animals. Human samples were scarce and environmental compartments were not included. Only by jointly analyzing isolates from humans, food and farm animals, and environmental sources such as water, soil, and environmental surfaces can a complete picture of transmission pathways be established. Future studies should address these shortcomings by including and sequencing genomes from isolates derived from non-food and environmental samples. In addition, the proposed species- and subspecies-level descriptions of the putative *A. lanthieri*-related species and the putative *A. butzleri* subspecies should be supported by further phenotypic characterization and comprehensive genomic analyses.

Collectively, our findings demonstrate that *Aliarcobacter* spp. from sub-Saharan African agricultural and human sources possess a dynamic accessory genome contributing to diverse virulence and resistance factors. The identification of potentially novel clades and multidrug-resistant lineages reinforces the need for integrated One Health approaches to monitor these pathogens. Understanding the genomic diversity and resistance mechanisms of *Aliarcobacter* is essential to inform public health risk assessments, improve diagnostic specificity, and guide antimicrobial stewardship in resource-limited settings.

## 5 Conclusions

This study underscores *Aliarcobacter*'s remarkable genomic plasticity, facilitating its persistence and potential cross-host transmission across human, animal, and environmental reservoirs. The convergence of extensive genetic diversity, AMR, and virulence factors highlights *Aliarcobacter* as an emerging public health concern. The lack of phylogeographic and host-specific clustering, coupled with the widespread presence of ARGs, suggests that environmental and agricultural factors may play a key role in its dissemination. These findings reinforce the urgent need for a One Health approach, integrating genomic, metagenomic, and ecological data to unravel transmission dynamics of *Aliarcobacter*, their role in AMR spread, and their broader impact on food safety and zoonotic infections.

## CRediT authorship contribution statement

**Andreas E. Zautner:** Writing – review & editing, Writing – original draft, Visualization, Validation, Conceptualization. **Ellis K. Paintsil:** Writing – review & editing, Writing – original draft, Visualization, Software, Investigation, Data curation. **Charity Wiafe Akenten:** Writing – review & editing, Supervision, Project administration, Investigation. **Stephan Lorenzen:** Writing – review & editing, Visualization, Software, Methodology, Investigation, Formal analysis, Data curation, Conceptualization. **Linda Aurelia Ofori:** Writing – review & editing, Investigation. **Joyce Mbwana:** Writing – review & editing, Investigation. **Neyaz Ahmed Khan:** Writing – review & editing, Investigation. **John Andrea Lusingu:** Writing – review & editing, Investigation. **Joseph Kaseka:** Writing – review & editing, Investigation. **Daniel Minja:** Writing – review & editing, Investigation. **Samwel Gesase:** Writing – review & editing, Investigation. **Anna Jaeger:** Writing – review & editing, Investigation. **Maike Lamshöft:** Writing – review & editing, Investigation. **Achim J. Kaasch:** Writing – review & editing, Resources, Methodology. **Kwasi Obiri-Danso:** Writing – review & editing, Investigation. **Ralf Krumkamp:** Writing – review & editing, Software, Methodology, Investigation. **Jürgen May:** Writing – review & editing, Supervision, Resources, Project administration, Methodology, Funding acquisition, Conceptualization. **Denise Dekker:** Writing – review & editing, Supervision, Resources, Project administration, Investigation, Funding acquisition, Conceptualization.

## Declaration of competing interest

The authors declare that they have no known competing financial interests or personal relationships that could have appeared to influence the work reported in this paper.

## Data Availability

Sequence data was already uploaded to NCBI GenBank.

## Appendix A Supplementary data

Supplementary Table 1. Overview of all isolates and genomes included in the study, along with their NCBI accession numbers.

Supplementary Table 2. Legend for the heatmap in Figure 6 indicating the presence of Aliarcobacter antimicrobial resistance genes, including the corresponding NCBI accession numbers and locus tags.

Supplementary Table 3. Legend for the heatmap in Figure 7 indicating the presence of Aliarcobacter virulence-associated genes, including the corresponding NCBI accession numbers and locus tags.
