# Supplementary material for: Genomic characterization of Aliarcobacter spp. from livestock in commercial and smallholder farms and from children in Ghana and Tanzania
Source: One Health. 2026 Oct 13;23:101616. doi: 10.1016/j.onehlt.2026.101616 (PMC13691151; doi:10.1016/j.onehlt.2026.101616)
Supplement: Supplementary file 1 [file mmc2.docx]

**Supplementary Table 2. *Aliarcobacter* antimicrobial resistance genes**

Legend for the heatmap in Figure 6 indicating the presence of *Aliarcobacter* antimicrobial resistance genes, including the corresponding NCBI accession numbers and locus tags.

| **locus tag** | **GenBank accession** | **strain** | **gene / putative protein function** |
| --- | --- | --- | --- |
| N3115_08190 | CP104332 | *A. butzleri* P1200 | *gyr*A (C254T) DNA gyrase subunit A |
| NGX11_11185 | CP099557 | *A. cryaerophilus* ICDCAC48 plasmid pCNAC48 | *optr*A ABC-F type ribosomal protection protein OptrA |
| ACNSON_05385 | CP183216 | *A. cryaerophilus* BNI-3540 | *ade*F efflux RND transporter permease subunit |
| N3114_03595 | CP104378 | *A. butzleri* P1100 | *ant(6)−Ia* aminoglycoside nucleotidyltransferase ANT(6)-Ia |
| ACNSON_10265 | CP183217 | *A. cryaerophilus* BNI-3540 plasmid p150-BNI-3540 | *aph(3)*−IIIa aminoglycoside O-phosphotransferase APH(3')-IIIa |
| ACNSON_10245 | CP183217 | *A. cryaerophilus* BNI-3540 plasmid p150-BNI-3540 | *aph(2'')−*Ia aminoglycoside O-phosphotransferase APH(2'')-Ia |
| N3114_05580 | CP104378 | *A. butzleri* P1100 | *aad*D aminoglycoside O-nucleotidyltransferase ANT(4')-Ia |
| ACN2C0_08620 | CP183402 | *A. butzleri* BNI-3560 | *aad*A2 aadA family aminoglycoside 3''-O-nucleotidyltransferase |
| CP183217 | CP183217 | *A. cryaerophilus* BNI-3540 plasmid p150-BNI-3540 | *aac*6 aminoglycoside N(6')-acetyltransferase type 1 |
| N3114_05575 | CP104378 | *A. butzleri* P1100 | *ble*O bleomycin binding protein |
| ACNSON_10260 | CP183217 | *A. cryaerophilus* BNI-3540 plasmid p150-BNI-3540 | *sat*−4 streptothricin N-acetyltransferase Sat4 |
| N3114_05600 | CP104378 | *A. butzleri* P1100 | *str* aminoglycoside adenylyltransferase family protein |
| N3114_03545 | CP104378 | *A. butzleri* P1100 | *cat*A type A chloramphenicol O-acetyltransferase |
| ACNSON_10765 | CP183217 | A. cryaerophilus BNI-3540 plasmid p150-BNI-3540 | *tet*Y tetracycline efflux MFS transporter Tet(Y) |
| N3114_05590 | CP104378 | *A. butzleri* P1100 | *tet*L tetracycline efflux MFS transporter Tet(L) |
| N3114_12525 | CP104379 | *A. butzleri* P1100 plasmid p63_DSM_105472 | *tet*M tetracycline resistance ribosomal protection protein Tet(M) |
| N3114_12585 | CP104379 | *A. butzleri* P1100 plasmid p63_DSM_105472 | *tet*A (*tetH*) tetracycline efflux MFS transporter Tet(H) |
| NGX11_11445 | CP099557 | *A. cryaerophilus* ICDCAC48 plasmid pCNAC48 | *ere*B EreB family erythromycin esterase |
| ACOAK2_11950 | CP184265 | *A. butzleri* BNI-3166 | *msr*D ABC-F type ribosomal protection protein Msr(D) |
| ACOAK2_11960 | CP184265 | *A. butzleri* BNI-3166 | *mef*A macrolide efflux MFS transporter Mef(A) |
| N3115_03275 | CP104332 | *A. butzleri* P1200 | *tol*C TolC family protein |
| N3115_03290 | CP104332 | *A. butzleri* P1200 | *mac*B2 ABC transporter permease |
| N3115_03285 | CP104332 | *A. butzleri* P1200 | *mac*B1 ABC transporter ATP-binding protein |
| N3114_03255 | CP104378 | *A. butzleri* P1100 | *macA* efflux RND transporter periplasmic adaptor subunit |
| P3286_07715 | CP120314 | *C. jejuni* PS 1 2021 | ermB 23S rRNA adenine(2058)-N(6))- methyltransferase Erm(B) |
| RJG54_03995 | CP134846 | *A. cryaerophilus* LEO 107 | *cme*R (*mar*R) winged helix DNA-binding protein |
| RJG54_03990 | CP134846 | *A. cryaerophilus* LEO 107 | *cme*C TolC family protein |
| RJG54_03980 | CP134846 | *A. cryaerophilus* LEO 107 | *cmeB* multidrug efflux RND transporter permease subunit |
| RJG54_03985 | CP134846 | *A. cryaerophilus* LEO 107 | *cem*A efflux RND transporter periplasmic adaptor subunit |
| NGX11_11130 | CP099557 | *A. cryaerophilus* ICDCAC48 plasmid pCNAC48 | *bla*_VEB_ VEB family extended-spectrum class A beta-lactamase |
| N3114_07545 | CP104378 | *A. butzleri* P1100 | *bla*_OXA−464_ class D beta−lactamase |
