# Supplementary material for: Genomic characterization of Aliarcobacter spp. from livestock in commercial and smallholder farms and from children in Ghana and Tanzania
Source: One Health. 2026 Oct 13;23:101616. doi: 10.1016/j.onehlt.2026.101616 (PMC13691151; doi:10.1016/j.onehlt.2026.101616)
Supplement: Supplementary file 2 [file mmc3.docx]

**Supplementary Table 3. *Aliarcobacter* virulence-associated genes**

Legend for the heatmap in Figure 7 indicating the presence of *Aliarcobacter* virulence-associated genes, including the corresponding NCBI accession numbers and locus tags.

| **locus tag** | **GenBank accession** | **strain** | **gene / putative protein function** |
| --- | --- | --- | --- |
| N3114_02290 | CP104378 | *A. butzleri* strain P1100 | *cad*F−like OmpA family protein |
| N3114_00335 | CP104378 | *A. butzleri* strain P1100 | *cj1349*−like OmpA outer membrane fibronectin-binding protein |
| N3114_04310 | CP104378 | *A. butzleri* strain P1100 | *pld*A phospholipase A1 |
| N3114_10790 | CP104378 | *A. butzleri* strain P1100 | *htr*A Do family serine endopeptidase |
| N3114_08075 | CP104378 | *A. butzleri* strain P1100 | *cia*B invasion protein CiaB |
| V7P26_07630 | CP146528 | *A. cryaerophilus* Kiel 2759 | *pgl*C undecaprenyl phosphate N,N'-diacetylbacillosamine 1-phosphate transferase |
| V7P26_07625 | CP146528 | *A. cryaerophilus* Kiel 2759 | *pgl*D UDP-N-acetylbacillosamine N-acetyltransferase |
| ACRYD_1248 | CP032825 | *A. cryaerophilus* D2610 | *pgl*E aminotransferase, DegT/DnrJ/EryC1/StrS family |
| QUR77_02095 | CP129947 | *A. cryaerophilus* DSM 115954 | *pgl*F UDP-N-acetylglucosamine 4,6-dehydratase (configuration-retaining) |
| ACRYD_1258 | CP032825 | *A. cryaerophilus* D2610 | *pse*A pseudaminic acid biosynthesis protein, putative PseA |
| QUR77_07660 | CP129947 | *A. cryaerophilus* DSM 115954 | *pse*B UDP-N-acetylglucosamine 4,6-dehydratase (inverting) |
| QUR77_07685 | CP129947 | *A. cryaerophilus* DSM 115954 | *pse*I pseudaminic acid synthase |
| ACRYD_0117 | CP032825 | *A. cryaerophilus* D2610 | *pse*F CMP-pseudaminic acid synthetase |
| ACRYD_0151 | CP032825 | *A. cryaerophilus* D2610 | *ptm*B N-acetylneuraminate synthase family protein,putative legionaminic acid synthase LegI |
| QUR79_05035 | CP129950 | *A. cryaerophilus* DSM 115972 | *che*Y Chemotaxis protein CheY |
| QUR79_05030 | CP129950 | *A. cryaerophilus* DSM 115972 | *flg*G flagellar basal-body rod protein FlgG |
| QUR79_05180 | CP129950 | *A. cryaerophilus* DSM 115972 | *flh*A flagellar biosynthesis protein FlhA |
| QUR79_05160 | CP129950 | *A. cryaerophilus* DSM 115972 | *flh*B flagellar biosynthesis protein FlhB |
| QUR79_05170 | CP129950 | *A. cryaerophilus* DSM 115972 | *fli*I flagellar protein export ATPase FliI |
| QUR79_05105 | CP129950 | *A. cryaerophilus* DSM 115972 | *fli*N FliM/FliN family flagellar motor switch protein |
| QUR79_07255 | CP129950 | *A. cryaerophilus* DSM 115972 | *fli*P flagellar type III secretion system pore protein FliP |
| QUR79_05495 | CP129950 | *A. cryaerophilus* DSM 115972 | *mot*A flagellar motor stator protein MotA |
| QUR79_05490 | CP129950 | *A. cryaerophilus* DSM 115972 | *mot*B flagellar motor protein MotB |
| QUR78_05380 | CP129948 | *A. cryaerophilus* DSM 115955 | *acr*A efflux RND transporter periplasmic adaptor subunit |
| QUR78_05375 | CP129948 | *A. cryaerophilus* DSM 115955 | *acr*B efflux RND transporter permease subunit |
| ACRYD_0291 | CP032825 | *A. cryaerophilus* D2610 | *eno* enolase |
| N3114_01895 | CP104378 | *A. butzleri* strain P1100 | *lplat*−like lysophospholipid acyltransferase family protein |
| ACRYD_0082 | CP032825 | *A. cryaerophilus* D2610 | *acp*XL/*lpx*A UDP-N-acetylglucosamine acyltransferase |
| QUR79_03935 | CP129950 | *A. cryaerophilus* DSM 115972 | *fab*Z 3-hydroxyacyl-ACP dehydratase FabZ |
| V7P26_07710 | CP146528 | *A. cryaerophilus* Kiel 2759 | *bpl*B acyltransferase |
| V7P26_04365 | CP146528 | *A. cryaerophilus* Kiel 2759 | *ton*B energy transducer TonB |
| V7P26_04370 | CP146528 | *A. cryaerophilus* Kiel 2759 | *exb*D/*tol*R TonB system transport protein ExbD |
| V7P26_04375 | CP146528 | *A. cryaerophilus* Kiel 2759 | *exb*B TonB-system energizer ExbB |
| V7P26_03435 | CP146528 | *A. cryaerophilus* Kiel 2759 | *fep*A TonB-dependent receptor |
| N3114_04885 | CP104378 | *A. butzleri* strain P1100 | *fcu*A2 TonB-dependent receptor |
| V7P26_04710 | CP146528 | *A. cryaerophilus* Kiel 2759 | *tbd*RI TonB family protein |
| N3114_05965 | CP104378 | *A. butzleri* strain P1100 | *tbd*RII TonB-dependent receptor |
| V7P26_06450 | CP146528 | *A. cryaerophilus* Kiel 2759 | *isd*F/*fhu*C helical backbone metal receptor |
| N3114_10120 | CP104378 | *A. butzleri* strain P1100 | *cir*1 TonB-dependent receptor |
| N3114_12100 | CP104378 | *A. butzleri* strain P1100 | *cir*2 TonB-dependent receptor |
| N3114_11110 | CP104378 | *A. butzleri* strain P1100 | *fcu*A1 TonB-dependent siderophore receptor |
| V7P26_04530 | CP146528 | *A. cryaerophilus* Kiel 2759 | *irg*A TonB-dependent receptor |
| Abu_0727 | CP000361 | *A. butzleri* RM4018 | *iro*E possible IroE protein |
| N3114_08725 | CP104378 | *A. butzleri* strain P1100 | *irp*A imelysin iron-regulated protein A precursor |
| QUR79_03770 | CP129950 | *A. cryaerophilus* DSM 115972 | *fut*A1 Fe(3+) ABC transporter substrate-binding protein |
| QUR79_03765 | CP129950 | *A. cryaerophilus* DSM 115972 | *cfbp*B iron ABC transporter permease |
| QUR79_03760 | CP129950 | *A. cryaerophilus* DSM 115972 | *cfbp*C ABC transporter ATP-binding protein |
| QUR79_07685 | CP129950 | *A. cryaerophilus* DSM 115972 | *fur* Fur family transcriptional regulator |
| QUR79_07675 | CP129950 | *A. cryaerophilus* DSM 115972 | *feo*B ferrous iron transport protein B |
| QUR79_00815 | CP129950 | *A. cryaerophilus* DSM 115972 | *fie*F cation diffusion facilitator family transporter |
| Abu_0940 | CP000361 | *A. butzleri* RM4018 | *hec*A adhesin/haemagglutinin, HecA family |
| Abu_0939 | CP000361 | *A. butzleri* RM4018 | *hec*B hemolysin activation protein HecB |
| V7P26_02790 | CP146528 | *A. cryaerophilus* Kiel 2759 | *tly*A (ftsJ-like) TlyA family rRNA (cytidine-2'-O)-methyltransferase |
| QUR79_03075 | CP129950 | *A. cryaerophilus* DSM 115972 | *his*F imidazole glycerol phosphate synthase subunit HisF |
| QUR79_06915 | CP129950 | *A. cryaerophilus* DSM 115972 | *pan*D aspartate 1-decarboxylase |
| AEBR_2804 | CP053836 | *Halarcobacter ebronensis* LMG 27922 | *dms*A anaerobic DMSO reductase DmsABC, chain A |
| AEBR_2805 | CP053836 | *Halarcobacter ebronensis* LMG 27922 | *dms*B anaerobic DMSO reductase DmsABC, chain B, iron-sulfur subunit |
| AEBR_2806 | CP053836 | *Halarcobacter ebronensis* LMG 27922 | *dms*C anaerobic DMSO reductase DmsABC, chain C, anchor subunit |
| QUR79_04770 | CP129950 | *A. cryaerophilus* DSM 115972 | *nap*A nitrate reductase catalytic subunit NapA |
| QUR77_07845 | CP129947 | *A. cryaerophilus* DSM 115954 | *neu*B2 N-acetylneuraminate synthase |
| QUR77_07835 | CP129947 | *A. cryaerophilus* DSM 115954 | *neu*C2 UDP-N-acetylglucosamine 2-epimerase |
| QUR77_07715 | CP129947 | *A. cryaerophilus* DSM 115954 | *gal*E UDP-glucose 4-epimerase GalE |
| N3114_03120 | CP104378 | *A. butzleri* strain P1100 | *gtr*B glycosyltransferase |
| QUR79_10025 | CP129950 | *A. cryaerophilus* DSM 115972 | *wbj*D /*wec*B UDP-N-acetylglucosamine 2-epimerase |
| QUR79_10020 | CP129950 | *A. cryaerophilus* DSM 115972 | *wec*C UDP-*N*-acetyl-D-mannosamine dehydrogenase |
| QUR77_07735 | CP129947 | *A. cryaerophilus* DSM 115954 | *gmd*−1 GDP-mannose 4,6-dehydratase |
| ACRYD_0132 | CP032825 | *A. cryaerophilus* D2610 | *ugd* UDP-glucose 6-dehydrogenase |
| QUR79_09880 | CP129950 | *A. cryaerophilus* DSM 115972 | *uge* NAD-dependent epimerase/dehydratase family protein |
| QUR77_09580 | CP129947 | *A. cryaerophilus* DSM 115954 | *rfb*C/*wbc*A dTDP-4-dehydrorhamnose 3,5-epimerase |
| V7P26_12805 | CP146528 | *A. cryaerophilus* Kiel 2759 | *gac*A response regulator transcription factor |
| V7P26_02970 | CP146528 | *A. cryaerophilus* Kiel 2759 | *wbt*A FdtA/QdtA family cupin domain-containing protein |
| V7P26_02985 | CP146528 | *A. cryaerophilus* Kiel 2759 | *wbt*I/*deg*T DegT/DnrJ/EryC1/StrS family aminotransferase |
| QT384_01980 | CP128652 | *A. cryaerophilus* DSM 115960 | *wbp*B Gfo/Idh/MocA family oxidoreductase |
| QT384_01970 | CP128652 | *A. cryaerophilus* DSM 115960 | *wbp*D WxcM-like sugar acyltransferase |
| QT384_01965 | CP128652 | *A. cryaerophilus* DSM 115960 | *wbp*E DegT/DnrJ/EryC1/StrS family aminotransferas |
| QUR79_09910 | CP129950 | *A. cryaerophilus* DSM 115972 | *wbp*G N-acetyl sugar amidotransferase |
| QT384_01985 | CP128652 | *A. cryaerophilus* DSM 115960 | *tvi*B nucleotide sugar dehydrogenas |
| QT384_01925 | CP128652 | *A. cryaerophilus* DSM 115960 | *wbp*I/*wec*B UDP-N-acetylglucosamine 2-epimerase (non-hydrolyzing) |
| QUR79_00950 | CP129950 | *A. cryaerophilus* DSM 115972 | *kat*E katalase |
| QUR79_00965 | CP129950 | *A. cryaerophilus* DSM 115972 | *sod* superoxide dismutase |
| QUR79_00690 | CP129950 | *A. cryaerophilus* DSM 115972 | *per*R Peroxide-responsive repressor PerR |
| N3114_12670 | CP104380 | *A. butzler*i P1100 plasmid pVir_DSM_105472 | *vir*B2 TrbC/VirB2 family protein |
| N3114_12680 | CP104380 | *A. butzler*i P1100 plasmid pVir_DSM_105472 | *vir*B4 type IV secretion system protein |
| N3114_12685 | CP104380 | *A. butzler*i P1100 plasmid pVir_DSM_105472 | *vir*B6 type IV secretion system protein |
| N3114_12695 | CP104380 | *A. butzler*i P1100 plasmid pVir_DSM_105472 | *vir*B8 type IV secretion system protein |
| N3114_12700 | CP104380 | *A. butzler*i P1100 plasmid pVir_DSM_105472 | *vir*B9 type IV secretion system protein |
| N3114_12705 | CP104380 | *A. butzler*i P1100 plasmid pVir_DSM_105472 | *vir*B10 type IV secretion system protein |
| N3114_12720 | CP104380 | *A. butzler*i P1100 plasmid pVir_DSM_105472 | *vir*B11 ATPase, T2SS/T4P/T4SS family |
| N3114_12730 | CP104380 | *A. butzler*i P1100 plasmid pVir_DSM_105472 | *vir*D4 type IV secretory system conjugative DNA transfer family protein |
